# Constructing Direct Z-Scheme Y_2_TmSbO_7_/GdYBiNbO_7_ Heterojunction Photocatalyst with Enhanced Photocatalytic Degradation of Acetochlor under Visible Light Irradiation

**DOI:** 10.3390/ijms25136871

**Published:** 2024-06-22

**Authors:** Liang Hao, Jingfei Luan

**Affiliations:** 1 School of Physics, Changchun Normal University, Changchun 130032, China; hliang0725@163.com; 2 State Key Laboratory of Pollution Control and Resource Reuse, School of the Environment, Nanjing University, Nanjing 210093, China

**Keywords:** Y_2_TmSbO_7_, GdYBiNbO_7_, Y_2_TmSbO_7_/GdYBiNbO_7_ heterojunction photocatalyst, direct Z-scheme, acetochlor, visible light exposure, photocatalytic activity, degradation pathway, degradation mechanism

## Abstract

This study presents a pioneering synthesis of a direct Z-scheme Y_2_TmSbO_7_/GdYBiNbO_7_ heterojunction photocatalyst (YGHP) using an ultrasound-assisted hydrothermal synthesis technique. Additionally, novel photocatalytic nanomaterials, namely Y_2_TmSbO_7_ and GdYBiNbO_7_, were fabricated via the hydrothermal fabrication technique. A comprehensive range of characterization techniques, including X-ray diffractometry, Fourier-transform infrared spectroscopy, Raman spectroscopy, UV-visible spectrophotometry, X-ray photoelectron spectroscopy, transmission electron microscopy, X-ray energy-dispersive spectroscopy, fluorescence spectroscopy, photocurrent testing, electrochemical impedance spectroscopy, ultraviolet photoelectron spectroscopy, and electron paramagnetic resonance, was employed to thoroughly investigate the morphological features, composition, chemical, optical, and photoelectric properties of the fabricated samples. The photocatalytic performance of YGHP was assessed in the degradation of the pesticide acetochlor (AC) and the mineralization of total organic carbon (TOC) under visible light exposure, demonstrating eximious removal efficiencies. Specifically, AC and TOC exhibited removal rates of 99.75% and 97.90%, respectively. Comparative analysis revealed that YGHP showcased significantly higher removal efficiencies for AC compared to the Y_2_TmSbO_7_, GdYBiNbO_7_, or N-doped TiO_2_ photocatalyst, with removal rates being 1.12 times, 1.21 times, or 3.07 times higher, respectively. Similarly, YGHP demonstrated substantially higher removal efficiencies for TOC than the aforementioned photocatalysts, with removal rates 1.15 times, 1.28 times, or 3.51 times higher, respectively. These improvements could be attributed to the Z-scheme charge transfer configuration, which preserved the preferable redox capacities of Y_2_TmSbO_7_ and GdYBiNbO_7_. Furthermore, the stability and durability of YGHP were confirmed, affirming its potential for practical applications. Trapping experiments and electron spin resonance analyses identified active species generated by YGHP, namely •OH, •O_2_^−^, and h^+^, allowing for comprehensive analysis of the degradation mechanisms and pathways of AC. Overall, this investigation advances the development of efficient Z-scheme heterostructural materials and provides valuable insights into formulating sustainable remediation strategies for combatting AC contamination.

## 1. Introduction

The application of pesticides in agricultural production has proven consequential due to the fact that it leads to an increase in crop yield per unit area, effectively addressing concerns such as food scarcity and reduced arable land resulting from population growth, industrialization, and rapid urbanization [1,2,3,4,5]. Acetochlor (AC), widely employed as a pre-emergence herbicide in major crops such as corn, peanuts, and soybeans, stands as one of the most extensively used herbicides worldwide [6,7,8,9]. However, the extensive usage of AC inevitably results in substantial AC residues in natural environments, entering water resources and accumulating within organisms through the food chain [10]. Furthermore, extensive research has exhibited the carcinogenic properties of AC, leading the United States Environmental Protection Agency to classify it as a B-2 carcinogen [8]. Consequently, an economically efficient technique for eliminating AC from wastewater has become an urgent necessity in terms of safeguarding the well-being of organisms and maintaining ecological equilibrium.

In the realm of water pollution treatments, conventional methods traditionally combine physical, chemical, and biological approaches, as evidenced in previous studies [11,12,13]. For instance, Xu et al. isolated and characterized four distinct microbial communities capable of degrading AC from soil and sludge samples contaminated with the compound [12]. Their experimental findings demonstrated that these mixed microbial communities were able to fully degrade AC at an initial concentration of 55 mg/L within a duration of four days. Additionally, Cai et al. utilized biological and chemical methods in their study to degrade AC. They employed organic fertilizer to enhance the activity of microorganisms in the soil, thereby facilitating the biodegradation of AC [11]. Furthermore, they utilized sodium thiosulfate as a modifier in their chemical degradation approach. Their results revealed that in soil modified with sodium thiosulfate, AC underwent a rapid degradation process, concluding with a final removal rate exceeding 85% over a 28-day period [11]. In a different approach, Wang et al. utilized a novel combination of physical and chemical methodologies for the degradation of AC [13]. In their experiment, the adsorption equilibrium of MnFe_2_O_4_-supported activated carbon magnetic adsorbent was achieved within 24 h, resulting in a greater than 90% removal rate of AC from the water [13]. Subsequently, they conducted an oxidation experiment using heat-activated ammonium supersulfate, which demonstrated a removal rate exceeding 90% for the AC absorbed to MnFe_2_O_4_-supported activated carbon magnetic adsorbent within 12 h [13]. However, in cycle experiments, the removal rate of the adsorbent was found to be limited to 50% [13]. Although these research studies have yielded promising outcomes in the degradation of acetochlor, it remains evident that traditional physical, chemical, and biological methods encounter inherent limitations, such as low efficiency, prolonged cycles, high costs, and the potential for secondary pollution when applied to water pollution treatment projects. Furthermore, industrial advancement has led to increasingly complex wastewater compositions, posing significant challenges for the effective degradation of organic pollutants. Consequently, more potent water treatment technologies must be developed to integrate with conventional approaches to degrade AC within the contaminated water resources.

Over the past few years, semiconductor photocatalysis has emerged as a feasible solution for water pollution treatment [14,15,16]. Photocatalysts, serving as the central component in photocatalytic technology, are capable of generating photoexcited carriers (PECs) through solar radiation [17,18]. These PECs could activate H_2_O and O_2_, giving rise to reactive oxygen species (•OH and •O_2_^−^) that effectively degrade pollutants [19,20]. While early research concentrated on metal oxide semiconductor photocatalysts, it was noted that they solely harness 4% of the solar spectrum, namely, ultraviolet light [21,22,23,24]. Consequently, researchers have turned their focus to A_2_B_2_O_7_-type photocatalysts, which exhibit noteworthy performance in terms of sunlight absorption and photocatalytic capabilities. For example, Zhang et al. reported on the photocatalytic removal of methyl orange using Gd_2_Ce_2_O_7_ powder and La_2_Ce_2_O_7_ powder under visible light exposure (VLTE) for 300 min [25]. The removal efficiencies achieved were 69.7% and 76.6%, respectively. Similarly, Yao et al. demonstrated that Gd_2_YSbO_7_ nanophase material achieved a substantial photocatalytic removal efficiency (REY) of 82.45% for benzotriazole under VLTE for 145 min [26].

In a previous study, the potential of structurally modifying a Bi_2_InTaO_7_ pyrochlore-structured photocatalyst was investigated [27]. Inspiration was drawn from prior research on TiO_2_, such as the work of Khan et al. [28], who synthesized Y-doped TiO_2_ and observed a significant enhancement in the photodegradation of methylene blue compared with pure TiO_2_, and also the research of Mazierski et al. [29], who synthesized Tm-doped TiO_2_ and observed a conspicuous improvement in the photodegradation of phenol in comparison to pristine TiO_2_. Zimbone et al. [30] additionally synthesized Sb-doped TiO_2_, which exhibited the enhanced degradation of methylene blue compared with undoped TiO_2_. Similarly, Xu et al. [31] synthesized Gd-doped TiO_2_, which demonstrated conspicuous improvements in the degradation of rhodamine B compared with undoped TiO_2_. Additionally, Sood et al. [32] synthesized Bi-doped TiO_2_, showing significant enhancements in the degradation of alizarin red S compared with undoped TiO_2_. Furthermore, Kong et al. [33] synthesized Nb-doped TiO_2_, demonstrating obvious enhancements in the degradation of acetaldehyde compared with undoped TiO_2_. The above studies collectively underscored the effectiveness of introducing Y, Tm, Sb, Gd, Bi, and Nb elements to enhance the photocatalytic activity of TiO_2_. Hence, by replacing certain elements in Bi_2_InTaO_7_, we hypothesized that the novel Y_2_TmSbO_7_ photocatalyst and GdYBiNbO_7_ photocatalyst possessed improved photocatalytic activity.

However, the challenge of high recombination rates among PECs remains prominent in photocatalysts based on single-component materials. Consequently, there is growing favor for binary heterostructure photocatalysts, where the interfaces between different components create intermediate electric fields that facilitate the separation of PECs. Currently, Z-scheme heterostructure photocatalysts have garnered more attention compared to traditional type-II heterojunction photocatalysts due to their superior photocatalytic activity [34,35,36,37,38].

Within the Z-scheme heterojunction system, photo-induced electrons and holes are retained within more negative conduction bands and more positive valence bands, respectively, while the futile PECs are recombined, introducing a greater redox potential than type-II systems. As a result, photocatalytic activity could be enhanced via the accelerated migration of PECs endowed with robust redox capabilities. Notably, conspicuous progress has been achieved in the field of Z-scheme heterojunction photocatalysts. For instance, Luo et al. rationally constructed a direct Z-scheme LaMnO_3_/g-C_3_N_4_ hybrid for improving the photocatalytic degradation efficiency of tetracycline under visible light conditions [39]. Lv et al. also prepared a direct Z-scheme CdS/Bi_4_V_2_O_11_ heterojunction catalyst with excellent performance towards the photodegradation of ciprofloxacin, tetracycline and rhodamine B [40]. Based on the above analysis, incorporating a Z-scheme heterojunction stands as an effective strategy in terms of facilitating PEC separation while significantly improving photocatalytic activity.

Inspired by these constructive insights and guided by appropriate band structures, we propose a novel direct Z-scheme Y_2_TmSbO_7_/GdYBiNbO_7_ heterojunction photocatalyst (YGHP) for the efficient removal of AC from pesticide wastewater under VLTE. This photocatalyst design harnesses a favorable Z-scheme heterojunction configuration to facilitate the separation of PECs, enhancing photocatalytic activity for AC degradation. The combination of Y_2_TmSbO_7_ and GdYBiNbO_7_ components in the heterojunction enables the effective utilization of visible light for the photocatalytic degradation process. The photodecomposing efficiency of the YGHP was experimentally validated, achieving an impressive AC removal rate of 99.75% after a mere 148 min of VLTE. This significant advancement highlights the superior efficacy of YGHP in comparison to other photocatalysts utilized for the degradation of AC, as demonstrated in Table S1 [7,41,42].

A comprehensive array of characterization techniques, including X-ray diffraction (XRD), UV-visible diffuse reflectance spectroscopy (UV-Vis DRS), Fourier-transform infrared spectroscopy (FTIR), Raman spectroscopy, X-ray photoelectron spectroscopy (XPS), transmission electron microscopy (TEM), energy-dispersive X-ray spectroscopy (EDS), photoluminescence spectroscopy (PL), photocurrent measurements, electrochemical impedance spectroscopy (EIS), ultraviolet photoelectron spectroscopy (UPS), and electron paramagnetic resonance (EPR) spectroscopy was employed to thoroughly analyze the morphological features, composition, chemical, optical, and photoelectric properties of pure-phase Y_2_TmSbO_7_ and GdYBiNbO_7_ photocatalysts. Furthermore, the photocatalytic degradation efficiency of AC in pesticide wastewater under VLTE was systematically evaluated for various photocatalysts, including the YGHP, individual Y_2_TmSbO_7_ and GdYBiNbO_7_ photocatalysts and N-doped TiO_2_. A major contribution of this study is the pioneering synthesis of visible-light-responsive Y_2_TmSbO_7_ and GdYBiNbO_7_ photocatalysts using the hydrothermal fabrication technique. Furthermore, the research achieved the first-time fabrication of a Z-type heterojunction photocatalyst composed of two A_2_B_2_O_7_ photocatalysts, resulting in high photodegradation performance using the ultrasound-assisted hydrothermal synthesis method. A series of photodegradation experiments were conducted to evaluate and compare the photocatalytic performance of the materials reported in our previous studies with the newly synthesized materials in this study. The experimental results are summarized in Table S2. Remarkably, Y_2_TmSbO_7_, GdYBiNbO_7_, and YGHP exhibited significantly improved photodegradation performance towards AC compared to other A_2_B_2_O_7_ photocatalysts and A_2_B_2_O_7_-based heterojunction photocatalysts previously reported. This finding highlights the conspicuous innovation and effectiveness of the YGHP in AC degradation, further emphasizing the significance of this study in the field of photocatalysis.

## 2. Results and Discussion

### 2.1. X-ray Diffraction Analysis

**Figure 1:**
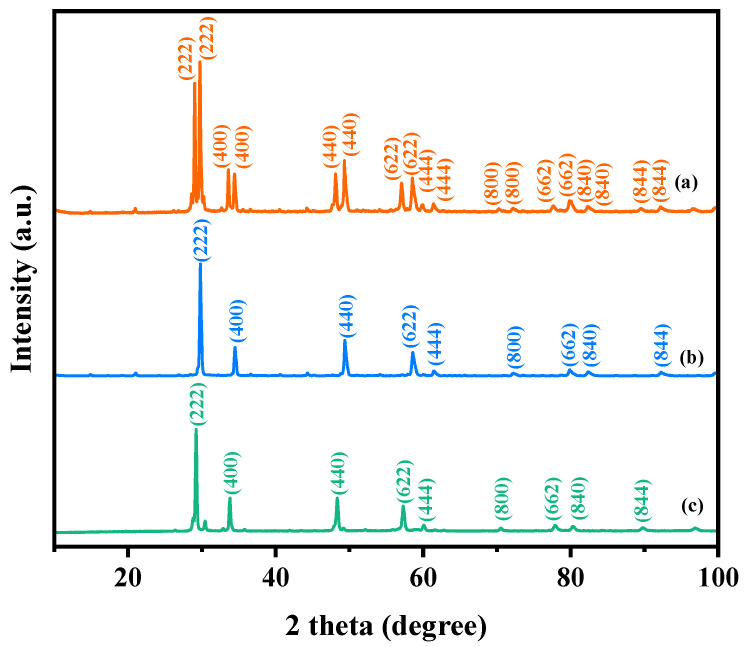
XRD images: (**a**) YGHP, (**b**) Y_2_TmSbO_7_, and (**c**) GdYBiNbO_7_.

**Figure 2:**
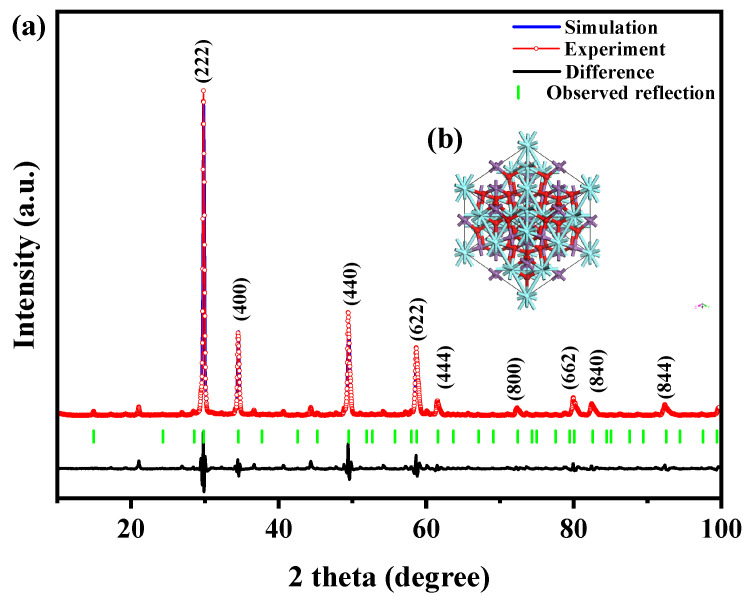
(**a**) XRD pattern and Rietveld refinement and (**b**) the atomic architecture (red atom: O; cyan atom: Y; purple atom: Tm or Sb) of Y_2_TmSbO_7_.

**Figure 3:**
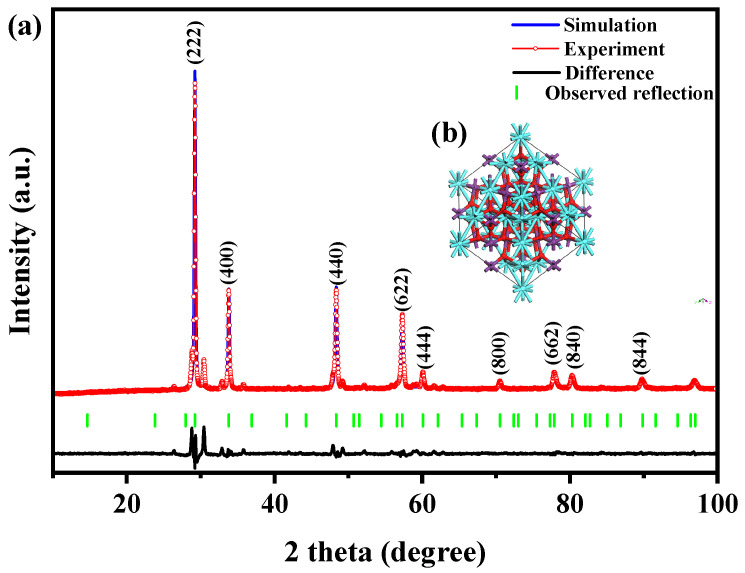
(**a**) XRD pattern and Rietveld refinement and (**b**) the atomic architecture (red atom: O; cyan atom: Gd or Y; purple atom: Bi or Nb) of GdYBiNbO_7_.

Figure 1 exhibits the X-ray diffraction profiles of the YGHP sample, Y_2_TmSbO_7_ sample, and the GdYBiNbO_7_ sample. The XRD data for the YGHP, Y_2_TmSbO_7_, and GdYBiNbO_7_ samples were specifically obtained from their respective XRD experiments. The XRD spectrum of YGHP includes all the peak and crystal surface indices found in the XRD spectra of Y_2_TmSbO_7_ and GdYBiNbO_7_, providing strong evidence for the successful synthesis of YGHP. To validate the crystallographic architecture, the X-ray diffraction data of Y_2_TmSbO_7_ and GdYBiNbO_7_ underwent Rietveld refinement using the Materials Studio program, as depicted in Figure 2a and Figure 3a, respectively [43]. The refinement outcomes for Y_2_TmSbO_7_ and GdYBiNbO_7_ yielded deviations of 5.11% and 4.01%, respectively, indicating an obvious concurrence between experimental and theoretical strengths [43], thereby validating the pyrochlore-type crystalline architecture of Y_2_TmSbO_7_ and GdYBiNbO_7_. Meanwhile, both compounds crystallize in the cubic crystallographic system with a space group of Fd3m, demonstrating single–phase behavior. The lattice constants were ascertained to be 10.377 Å for Y_2_TmSbO_7_ and 10.391 Å for GdYBiNbO_7_. The refinement model, accounting for the presence of oxygen atoms, provided a high level of agreement with the experimental strengths.

The atomic architectures of Y_2_TmSbO_7_ and GdYBiNbO_7_ are depicted in Figure 2b and Figure 3b, respectively. These architectures were erected according to the corresponding space group, crystal system, lattice constants, atomic coordinates, and structural parameters. Table 1 and Table 2 present comprehensive analyses of atomic coordinates and structural parameters for Y_2_TmSbO_7_ and GdYBiNbO_7_. These analyses serve as compelling validation for the configurable stableness of samples and highlight their potential as effectual photocatalytic materials in the water resource environment industry.

The distortions observed in the M(1)O_6_ octahedra (M(1) = Tm^3+^ and Sb^5+^) of Y_2_TmSbO_7_ signify a distortion in the crystal architecture, a phenomenon acknowledged for its ability to enhance photocatalytic efficiency in various previous studies [27,44]. The distinctive crystal architecture of Y_2_TmSbO_7_, characterized by interconnected M(1)O_6_ octahedra through Y^3+^ ions, highlights unique Y–O interatomic distances and angular inclinations. Particularly, the crystal architecture of Y_2_TmSbO_7_ displays two types of Y–O bonds: six longer Y–O(1) bonds (4.766 Å) and two shorter Y–O(2) bonds (2.242 Å) [27,44]. The interatomic distances of M(1)–O(1) were measured at 1.988 Å, while the interatomic distance of M(1)–Y was found to be 3.661 Å. The angular inclinations of M(1)–O–M(1) and Y–M(1)–Y were determined to be 134.020° and 135.00°, respectively. Additionally, the Y–M(1)–O angular inclinations were calculated to be 137.431°. These M(1)–O–M(1) angular inclinations in Y_2_TmSbO_7_ influence the locomotion of PECs and their capability to reach surface catalytic sites, thereby impacting photocatalytic effectiveness. Furthermore, the larger Y–Tm–O and Y–Sb–O angular inclinations in Y_2_TmSbO_7_ contribute further to its photocatalytic efficacy [27,44].

Likewise, in the crystal architecture of GdYBiNbO_7_, there existed two types of A–O bonds (A = Gd^3+^ and Y^3+^): six longer A–O(1) bonds (4.766 Å) and two shorter A–O(2) bonds (2.242 Å) [27,44]. The interatomic distances for M(2)–O(1) (M(2) = Bi^3+^ and Nb^5+^) were 1.988 Å, whereas for M(2)–A, it were 3.661 Å. The angular inclinations for M(2)–O–M(2) and A–M(2)–A were both 134.020° and 135.00°, respectively, in GdYBiNbO_7_. The A–M(2)–O angular inclinations were found to be 130.613°.

### 2.2. FTIR Analysis

**Figure 4:**
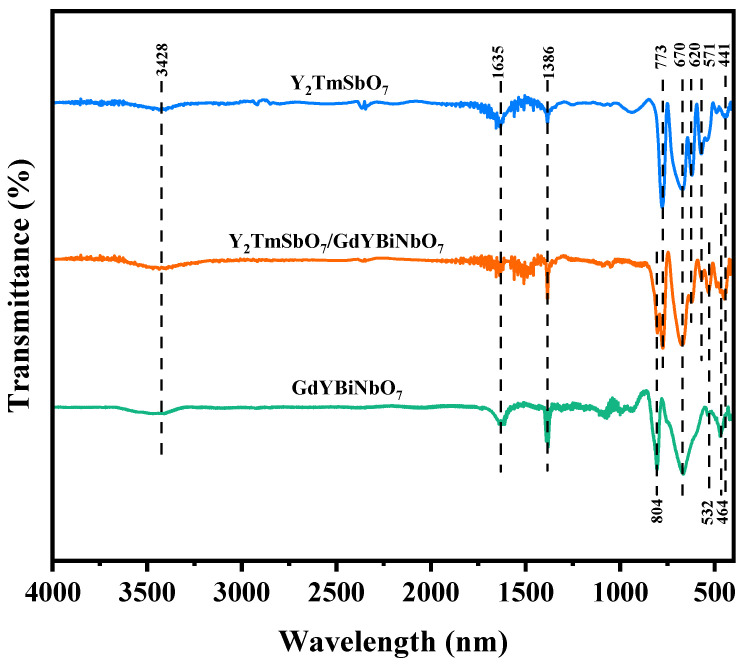
FTIR spectra of Y_2_TmSbO_7_, GdYBiNbO_7_, and YGHP.

FTIR spectroscopy was employed to elucidate information about the type and structure of chemical bonds in the YGHP, Y_2_TmSbO_7_ and GdYBiNbO_7_ samples, as illustrated in Figure 4. In the FTIR spectra of Y_2_TmSbO_7_ and GdYBiNbO_7_, distinctive stretching oscillations of the Bi–O bond were observed at a wavenumber of 464 cm^−1^ [45]. Similarly, bending oscillations of Gd–O were detected at 532 cm^−1^ [46]. Furthermore, bending oscillations of Y–O were observed at 670 cm^−1^, and an additional band associated with Y–O appeared at 441 cm^−1^ [47,48]. The band centered at 804 cm^−1^ could be ascribed to Nb–O–Nb bridging and Nb–O stretching [49]. Additionally, the stretching oscillations of the Tm–O bond were assigned to the band at 620 cm^−1^, while those associated with Sb–O–Sb bonds could be observed at 571 cm^−1^ and 773 cm^−1^ [50,51]. Moreover, the FTIR spectra exhibited a broad spectral absorption band centered at 3428 cm^−1^, which could be attributed to the valence oscillation of water molecules [52]. Another absorption band at 1635 cm^−1^ was identified as the characteristic stretch of the H–O–H bond [52]. Additionally, peaks observed at 1386 cm^−1^ were associated with the vibrational mode of C–H bonds [53]. Collectively, these findings provided compelling validation of the existence and stableness of the heterostructure between Y_2_TmSbO_7_ and GdYBiNbO_7_, yielding important insights for further understanding and application of these materials.

### 2.3. Raman Analysis

**Figure 5:**
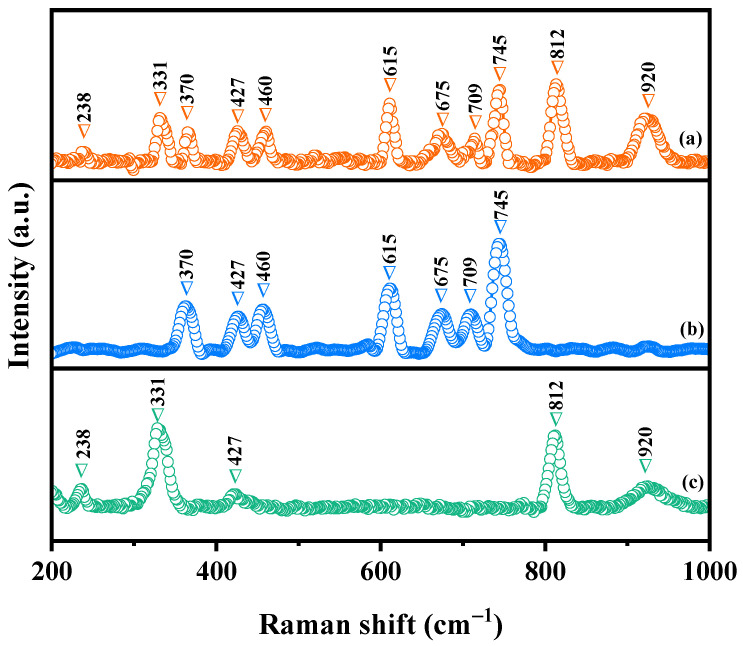
Raman spectra of (**a**) YGHP, (**b**) Y_2_TmSbO_7_, and (**c**) GdYBiNbO_7_.

Raman spectroscopy was utilized to probe the molecular structure and vibrational properties exhibited within the fabricated samples. Figure 5 illustrates the Raman spectra obtained for Y_2_TmSbO_7_, GdYBiNbO_7_, and YGHP. Significant modes were observed within the spectra of Y_2_TmSbO_7_ and GdYBiNbO_7_. The peak at 238 cm^−1^ corresponds to the bending vibration of the Nb–O–Nb bond, while the most prominent peak at 331 cm^−1^ was associated with the O–Gd–O bond, comprising a combination of the E_g_ mode and F_g_ mode [54,55]. Furthermore, the E_g_ mode and F_g_ mode of the Y–O bond were observed at 427 cm^−1^ [56]. The strong peak at 812 cm^−1^ was assigned to Nb–O–Nb chains in the [NbO_6_] octahedral [57]. Additionally, the peak at 920 cm^−1^ could be attributed to the stretching oscillation of Bi–O bonds within the BiO_6_ octahedra [58]. The strongest peak at 370 cm^−1^ was attributed to the A_g_ mode or the combination mode of A_g_ and F_g_ mode of the Tm–O bond [59]. Moreover, the peak at 460 cm^−1^ was attributed to the A_1_ mode of Sb-O-Sb bond, while the peaks at 615 cm^−1^, 675 cm^−1^, 709 cm^−1^, and 745 cm^−1^ were assignable to Sb–O stretching oscillation [60,61]. These observed peaks confirm the presence of pure phases, which are consistent with the XRD results. Distinct peaks were found in the Raman spectrum of YGHP at 238 cm^−1^, 331 cm^−1^, 370 cm^−1^, 427 cm^−1^, 460 cm^−1^, 615 cm^−1^, 675 cm^−1^, 709 cm^−1^, 745 cm^−1^, 812 cm^−1^, and 920 cm^−1^, indicative of the integration of the unique characteristics inherent in both Y_2_TmSbO_7_ and GdYBiNbO_7_. This observation further reinforces the successful fabrication of the heterostructure photocatalyst.

### 2.4. TEM-EDS Analysis

**Figure 6:**
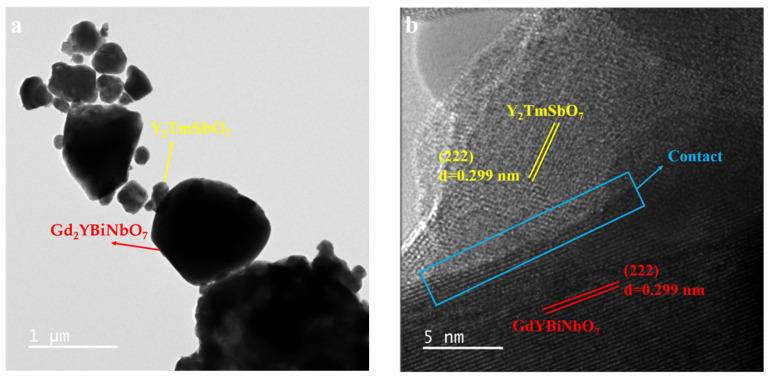
(**a**) TEM image and (**b**) HRTEM image of YGHP.

**Figure 7:**
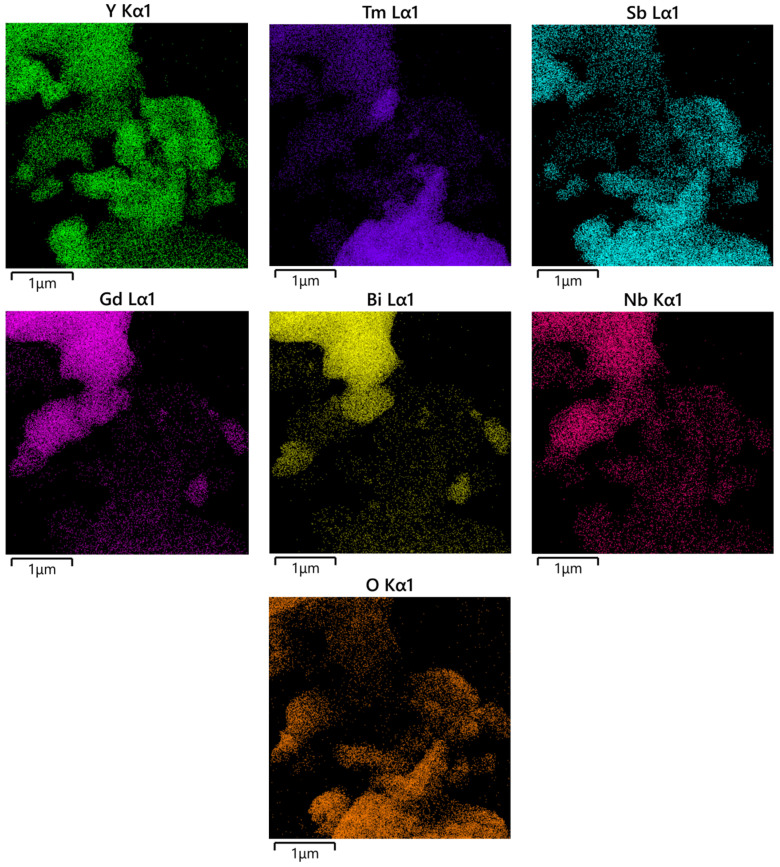
EDS elemental mapping of YGHP (Y, Tm, Sb, and O from Y_2_TmSbO_7_ and Gd, Y, Bi, Nb, and O from GdYBiNbO_7_).

**Figure 8:**
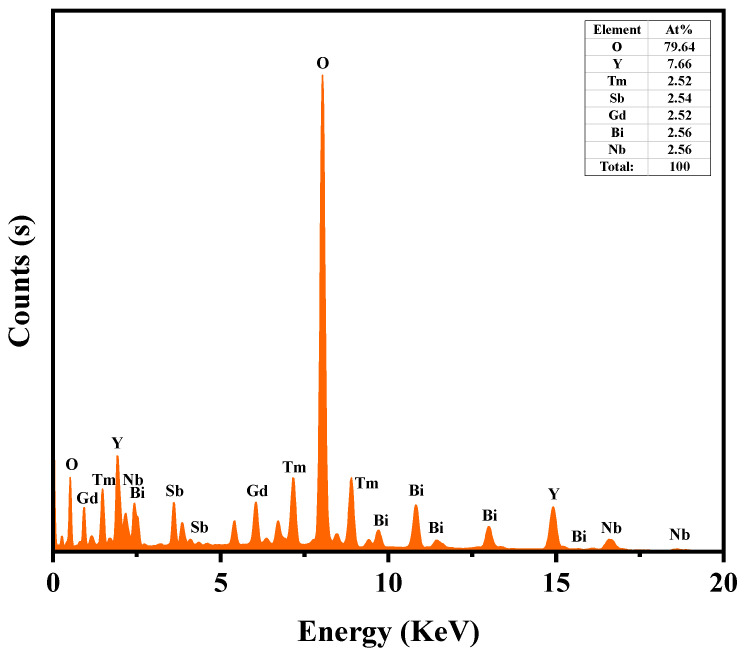
The EDS spectrum of YGHP.

Transmission electron microscopy (TEM) was used to examine the morphology of YGHP, and the elemental composition was determined using energy-dispersive X-ray spectroscopy (EDS). Figure 6 presents the TEM images and HRTEM images, while Figure 7 displays the EDS elemental mapping of YGHP. In Figure 6a, the TEM images clearly depicted the presence of Y_2_TmSbO_7_ nanoparticles along with GdYBiNbO_7_ nanoparticles within the YGHP composites. Furthermore, Figure 6b reveals the HRTEM image of the interface of the Y_2_TmSbO_7_ nanoparticles and GdYBiNbO_7_ nanoparticles marked in Figure 6a, clearly showing the interface between Y_2_TmSbO_7_ and GdYBiNbO_7_ and two distinct lattice stripes. The lattice spacing calculations yielded 0.299 nm for the (222) planes of Y_2_TmSbO_7_ and GdYBiNbO_7_, respectively. The findings shown in Figure 7 underscore the compelling evidence garnered through the EDS element mapping analysis, affirming the co-occurrence of Y, Tm, Sb, Gd, Bi, Nb, and O elements within the YGHP specimen. This substantiates the concurrent existence of both Y_2_TmSbO_7_ and GdYBiNbO_7_ compounds. Moreover, upon scrutinizing the light-emitting regions related to Tm and Sb in contrast to Gd, Bi, and Nb, it could be inferred that GdYBiNbO_7_ corresponds to the larger microboulders, whereas Y_2_TmSbO_7_ corresponds to the smaller microboulders. Additionally, the EDS spectrum shown in Figure 8 reveals an atomic ratio of around 766:252:254:252:256:256:7964 for the elements Y, Tm, Sb, Gd, Bi, Nb, and O. Based on the extensive findings above, it is evident that YGHP was effectively manufactured with eximious purity using the prescribed preparation parameters.

### 2.5. X-ray Photoelectron Spectroscopy Analysis

**Figure 9:**
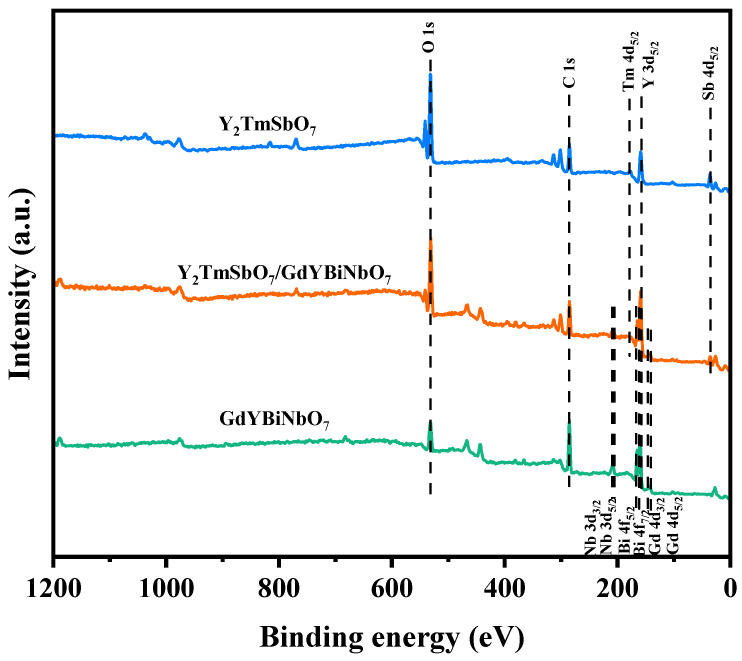
The full XPS spectrum of synthesized YGHP, Y_2_TmSbO_7_, and GdYBiNbO_7_.

**Figure 10:**
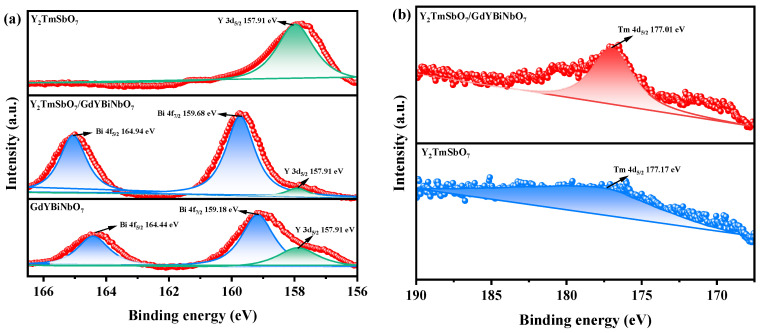

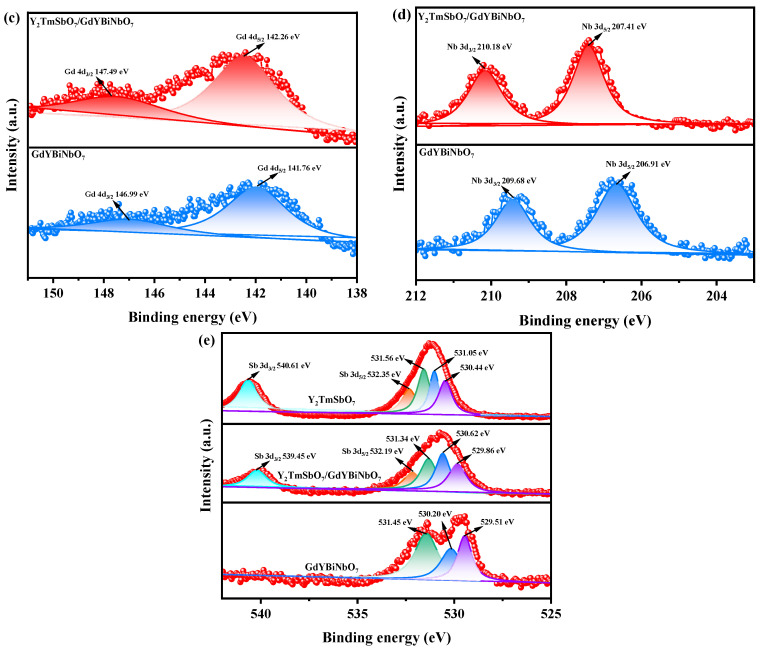
The high-resolution XPS spectra of (**a**) Y 3d and Bi 4f; (**b**)Tm 4d; (**c**) Gd 4d; (**d**) Nb 3d, and (**e**) O 1s and Sb 3d of YGHP, Y_2_TmSbO_7_, and GdYBiNbO_7_.

In this study, XPS was employed to investigate the surface chemical composition and electronic states of the samples, as demonstrated in Figure 9. The survey spectrum showed the detection of Y, Tm, Sb, Gd, Bi, Nb, and O elements in YGHP, with the carbon peak serving as a calibration reference. It was observed that Gd, Bi, and Nb signals were distinctly visible in YGHP in comparison to Y_2_TmSbO_7_, indicating the presence of GdYBiNbO_7_ in YGHP. The spectral peaks corresponding to Y 3d_5/2_, Bi 4f_7/2_, Bi 4f_5/2_, Tm 4d_5/2_, Gd 4d_5/2_, Nb 3d_5/2_, and Nb 3d_3/2_ in Y_2_TmSbO_7_, GdYBiNbO_7_, and YGHP are presented in Figure 10a–d. The peaks at 159.68 eV and 164.94 eV in the Bi 4f spectrum of YGHP were assigned to Bi 4f_7/2_ and Bi 4f_5/2_ states of Bi^3+^ [35,62,63]. This was confirmed by the measured spin-orbit separation value of 5.26 eV. However, the corresponding peaks in the GdYBiNbO_7_ spectrum were centered at lower binding energies, specifically at 159.18 eV and 164.44 eV, demonstrating a slight shift relative to YGHP. The Tm 4d_5/2_ peaks in YGHP shifted to lower binding energies, as demonstrated in Figure 10b, with the peaks centered at 177.01 eV, in contrast to those in Y_2_TmSbO_7_. Similarly, in Figure 10c, the peaks observed at 142.26 eV and 147.49 eV in the Gd 4d spectrum of YGHP were assigned to Gd 4d_5/2_ and Gd 4d_3/2_ states of Gd^3+^, respectively, as confirmed by the measured spin-orbit separation value of 5.23 eV [35,62,63]. In the GdYBiNbO_7_ spectrum, the corresponding peaks were centered at slightly lower binding energies, specifically 141.76 eV and 146.99 eV, reflecting a consistent shift relative to YGHP. In Figure 10d, the peaks observed at 207.41 eV and 210.18 eV in the Nb 3d spectrum of YGHP were assigned to Nb 3d_5/2_ and Nb 3d_3/2_ states of Nb^5+^, respectively, as confirmed by the measured spin-orbit separation value of 2.77 eV [35,62,63]. The corresponding peaks in the GdYBiNbO_7_ spectrum were also at slightly lower binding energies, specifically 206.91 eV and 209.68 eV. The deconvoluted O 1s spectrum of YGHP, Y_2_TmSbO_7_ and GdYBiNbO_7_ is displayed in Figure 10e. It exhibited distinct peaks at 529.86 eV, 530.44 eV, and 529.51 eV, indicative of lattice oxygen [64]. Additionally, peaks were observed at 530.62 eV, 531.05 eV, and 530.20 eV, suggesting the existence of hydroxyl groups [65]. Finally, the presence of oxygen vacancies was suggested by peaks at 531.34 eV, 531.56 eV, and 531.45 eV [64,65]. Furthermore, the binding energies of the Sb 3d_5/2_ and Sb 3d_3/2_ peaks in YGHP were lower than those in Y_2_TmSbO_7_, measured to be 532.19 eV and 539.45 eV, respectively, indicating the valence state of +5 for Sb [35,62,63]. The higher binding energies of Gd 4d, Bi 4f, and Nb 3d in YGHP compared to GdYBiNbO_7_, as well as the lower binding energies of Tm 3d and Sb 3d in YGHP compared to Y_2_TmSbO_7_, were attributed to the low electron density of Gd, Bi, and Nb, and the high electron density of Tm and Sb in YGHP [35,36].

Upon thorough analysis, the oxidation states of the ions Y, Tm, Sb, Gd, Bi, Nb, and O within the material were ascertained as +3, +3, +5, +3, +3, +5, and −2, respectively. Additionally, analysis of the surface elements disclosed an average atomic ratio of Y/Tm/Sb/Gd/Bi/Nb/O, approximately 764:253:254:254:255:255:7965, demonstrating a close alignment with the ratios obtained from the EDS analysis. The atomic proportions of Y/Tm/Sb and Gd/Y/Bi/Nb in the YGHP sample were determined to be 2.01:1.00:1.00 and 1.00:1.00:1.00:1.00, respectively. This verification confirms the precise production of the photocatalysts in line with the specified chemical formula. The XPS peak analysis of YGHP, Y_2_TmSbO_7_ and GdYBiNbO_7_ did not reveal the presence of any additional phases. In conclusion, these XPS observations provided valuable insights into the presence of a Z-scheme heterostructure in YGHP and further confirmed the strong chemical interaction between Y_2_TmSbO_7_ and GdYBiNbO_7_, supporting the XRD, FTIR, Raman, TEM, HRTEM, and EDS results.

### 2.6. UV-Vis Diffuse Reflectance Spectra

**Figure 11:**
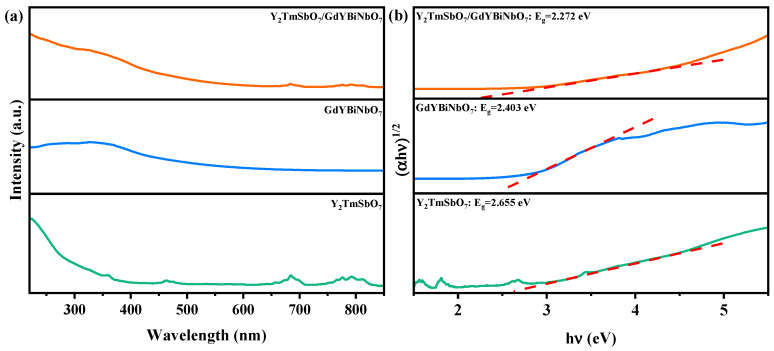
(**a**) The UV-Vis diffuse reflectance spectra and (**b**) correlative diagram of (*αhν*)^1/2^ and *hν* of the fabricated YGHP, Y_2_TmSbO_7_, and GdYBiNbO_7_.

To thoroughly examine the electronic band structure of the fabricated specimens, a meticulous examination of the diffused reflection absorption spectra for Y_2_TmSbO_7_, GdYBiNbO_7_, and YGHP was performed, as illustrated in Figure 11a. The onset of absorption for Y_2_TmSbO_7_ and GdYBiNbO_7_ was detected at wavelengths of approximately 450 nm and 520 nm, respectively. In contrast, YGHP displayed a unique absorption onset at around 550 nm, indicating a substantial shift towards longer wavelengths when compared to Y_2_TmSbO_7_ and GdYBiNbO_7_. This result implies that YGHP has a superior capacity for light absorption relative to Y_2_TmSbO_7_ and GdYBiNbO_7_.

To measure the energy gap between the electronic bands in these specimens, the Kubelka–Munk function (Equation (1)) was employed [66,67]. This method involves identifying the point where the photon energy (*hν*) axis intersects with the line drawn from the linear portion of the diffused reflection spectrum peaks. Using this approach, the energy difference between the bands in the specimens could be established.

(1) $$\frac{{\left[1-R_{d}(h\nu )\right]}^{2}}{2R_{d}(h\nu )}=\frac{\alpha (h\nu )}{S}$$

In the provided equation, we denote the scattering index, diffuse reflection, and the absorbance factor of radiation as *S*, *R_d_*, and *α*, respectively.

The optical absorption properties near the edges of the bands in the specimens aligned with Equation (2) [68,69]:

(2) (αhν)1n=A(hν−Eg)

In this equation, the variables *A*, *α*, *E_g_*, and *ν* denote the relative factor, absorbance factor, band energy difference, and photon frequency, respectively. Furthermore, parameter “*n*” is employed to characterize the type of electron transition following photoexcitation, with a value of 1/2 denoting direct transition and 2 indicating indirect transition [70]. After analyzing the data depicted in Figure 11b, we obtained the band gap measurements for Y_2_TmSbO_7_ and GdYBiNbO_7_ as 2.655 eV and 2.403 eV, respectively. The *n* value for both specimens was found to be approximately 2, suggesting that they underwent an indirect transition. Likewise, the analysis of YGHP yielded a band gap value of 2.272 eV, indicating an indirect transition for this material. This underscores the significantly lower band gap of YGHP compared to Y_2_TmSbO_7_ and GdYBiNbO_7_ and the higher light absorption capacity.

The *E_g_* value observed in YGHP, compared to that of Y_2_TmSbO_7_ and GdYBiNbO_7_, was likely attributable to the interface effects and electronic transfer behavior that occurred during the formation of the heterojunction. It was reasonable to consider that the inherent disparities in the properties of Y_2_TmSbO_7_ and GdYBiNbO_7_, upon interfacial interaction and formation within YGHP, could have resulted in altered charge distribution, band bending, and energy level shifts at the interface [71,72]. This might have played a role in influencing the electronic band structure. Consequently, the band structure was modified, resulting in a reduced bandgap width. Furthermore, electron charge migration behavior likely took place at the interface of YGHP due to the contrasting electron affinities and ionization potentials of its constituent components [72,73]. The charge transfer behavior could cause energy level shifts in the corresponding bands, further contributing to the band narrowing. As a consequence, YGHP showed a narrower bandgap compared to the individual Y_2_TmSbO_7_ and GdYBiNbO_7_. This bandgap-reducing effect had beneficial implications, such as broadening the absorption range of the heterojunction photocatalytic materials, facilitating the effective dissociation and transportation of optically generated electron–hole pairs, and enhancing its photocatalytic activity.

### 2.7. Photocatalytic Activity

**Figure 12:**
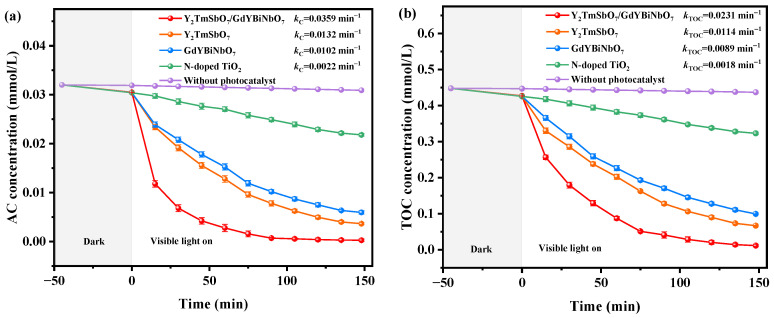
Saturation fluctuation charts of (**a**) AC and (**b**) TOC during photodegradation of AC with YGHP, Y_2_TmSbO_7_, GdYBiNbO_7_, N-T or without photocatalyst as the catalytic sample under VLTE.

The aim of this study was to evaluate the variability in AC concentration under VLTE conditions. This investigation employed several advanced photocatalysts, namely YGHP, Y_2_TmSbO_7_, GdYBiNbO_7_, and N-doped TiO_2_ (N-T). As depicted in Figure 12a, the data robustly illustrate a consistent reduction in AC levels upon exposure to these catalysts, thereby confirming their significant photodegradation capabilities. Conversely, control experiments involving only photolysis without these catalysts showed no significant change in AC concentration, unequivocally demonstrating that the photodegradation is primarily due to photocatalytic activities of these photocatalysts rather than mere photolysis [74,75].

To evaluate the removal efficiencies of AC, the formula ($1-\frac{C}{C_{0}}$) × 100% was employed, where *C* represents the concentration of AC at any given time during the photodegradation process, and *C*_0_ is its initial concentration. Data analysis from Figure 12a revealed that YGHP resulted in a significant REY of 99.75% in agrochemical sewage after 148 min under VLTE. This was associated with a reaction velocity (RV) of 3.59 × 10^−9^ mol·L^−1^·s^−1^ and a photonic efficiency (PEY) of 0.0755%. In contrast, using Y_2_TmSbO_7_ as the photocatalyst yielded an AC REY of 89.06%, with a RV of 3.21 × 10^−9^ mol·L^−1^·s^−1^ and a PEY of 0.0674%. Similarly, employing GdYBiNbO_7_ resulted in an AC REY of 82.19%, with a RV of 2.96 × 10^−9^ mol·L^−1^·s^−1^ and a PEY of 0.0622%. In contrast, the use of N-T achieved a lower AC REY of 32.50%, with a RV of 1.17 × 10^−9^ mol·L^−1^·s^−1^ and a PEY of 0.0246%.

These findings unequivocally indicate that among the photocatalysts examined, YGHP showed the most effective photodegradation of AC. The performances of Y_2_TmSbO_7_ and GdYBiNbO_7_ were also notably superior to that of N-T. After 148 min under VLTE, a comparison of the RV of AC revealed YGHP’s eximious performance, surpassing that of Y_2_TmSbO_7_ by 1.12 times, GdYBiNbO_7_ by 1.21 times, and N-T by 3.07 times.

Figure 12b illustrates the variance in total organic carbon (TOC) saturation during the photodecomposition of AC in agrochemical sewage under VLTE, utilizing various photocatalysts. The REY for TOC was calculated using the formula ($1-\frac{TOC}{TOC_{0}}$) × 100%, where *TOC* represents the instantaneous concentration of total organic carbon and *TOC*_0_ its initial concentration. Analysis of Figure 12b shows that, after 148 min of VLTE, the REY for TOC were 97.90%, 85.16%, 77.83%, or 28.42% for YGHP, Y_2_TmSbO_7_, GdYBiNbO_7_, or N-T, respectively. A comparative analysis of TOC mineralization effectiveness post-148 min under VLTE underscored the superior performance of YGHP, exceeding Y_2_TmSbO_7_ by 1.15 times, GdYBiNbO_7_ by 1.28 times, and N-T by 3.51 times.

Meanwhile, the first-order kinetic constants for AC and TOC degradation during the photodegradation process under VLTE, using various photocatalysts, including YGHP, Y_2_TmSbO_7_, GdYBiNbO_7_, and N-T, are shown in Figure 12 as well. The standard formulas ($ln\frac{C_{0}}{C}=k_{C}t)$ and ($ln\frac{{TOC}_{0}}{TOC}=k_{TOC}t)$ were utilized to calculate the kinetic constants of AC and TOC, respectively, where *C* and *C*_0_ denote the interim and initial concentrations of AC, and *TOC* and *TOC*_0_ represent the interim and initial concentrations of total organic carbon during the photodegradation process. The rate constants *k_C_* for AC degradation, as derived from the plots depicting dynamic saturation as a function of light exposure time, were measured as being 0.0359 min^−1^, 0.0132 min^−1^, 0.0102 min^−1^, and 0.0022 min^−1^ for YGHP, Y_2_TmSbO_7_, GdYBiNbO_7_, and N-T, respectively. A comparative analysis of the rate constants *k_C_* underscored the superior photodegradation performance of YGHP, exceeding Y_2_TmSbO_7_ by 2.72 times, GdYBiNbO_7_ by 3.52 times, and N-T by 16.32 times. In a similar investigation, the rate constants *k_TOC_* of TOC photodegradation were identified as being 0.0231 min^−1^, 0.0114 min^−1^, 0.0089 min^−1^, and 0.0018 min^−1^. A comparative analysis of *k_TOC_* underscored the superior mineralization performance of YGHP, exceeding Y_2_TmSbO_7_ by 2.03 times, GdYBiNbO_7_ by 2.60 times, and N-T by 12.83 times. Notably, the *k_TOC_* values were observed consistently to be inferior to the *k_C_* values for all catalysts, indicating the production of intermediate photodegradation products. Among the catalysts, YGHP demonstrated a significantly enhanced mineralization efficiency for AC degradation, which is a critical indicator of its superior performance as a photocatalyst.

The results unambiguously highlight YGHP’s eximious performance in effectively removing both AC and TOC during the photodecomposition process, surpassing Y_2_TmSbO_7_, GdYBiNbO_7_, and N-T by a significant margin. Notably, the TOC REY utilizing Y_2_TmSbO_7_ significantly exceeded that achieved with GdYBiNbO_7_ or N-T. These results emphasize the outstanding performance of YGHP in both AC mineralization and photodecomposition, positioning it as an exceedingly effective photocatalyst when compared to Y_2_TmSbO_7_, GdYBiNbO_7_, and N-T.

**Figure 13:**
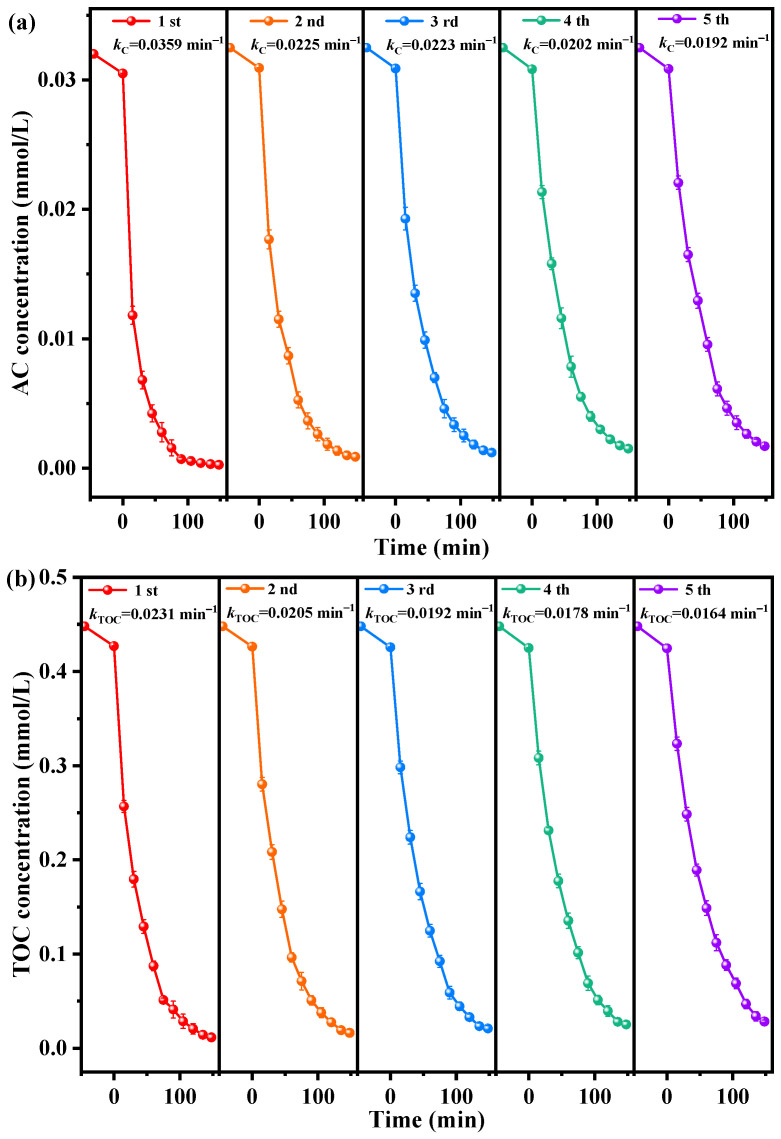
Saturation fluctuation images of (**a**) AC and (**b**) TOC during photodegradation of AC in pesticide-containing wastewater with YGHP as a photocatalyst under VLTE for successive degradation trials.

As shown in Figure 13a,b, the images depicting the saturation undulation of AC and TOC over five successive degradation cycles when YGHP was employed as a photocatalyst under VLTE demonstrate the structural stableness of YGHP and its potential for efficient reusability in photodecomposition processes [76]. Following 148 min of VLTE, the AC removal efficiencies recorded were 99.75%, 98.63%, 97.56%, 96.56%, and 95.63% over the cycles, respectively. Similarly, TOC removal efficiencies were 97.90%, 96.24%, 95.01%, 93.83%, and 92.45% across the respective cycles. These results affirm the recyclability and stability of YGHP over multiple degradation cycles, underscoring its potential for practical applications in sewerage treatment processes.

Meanwhile, the first-order kinetic constants for AC and TOC during the photocatalytic degradation of AC utilizing YGHP over five successive degradation cycles under VLTE condition are also shown in Figure 13. The kinetic constants *k_C_* for AC photodegradation, obtained from the plots depicting dynamic saturation as a function of light exposure time, consistently yielded high values, with calculations of 0.0359 min^−1^, 0.0225 min^−1^, 0.0223 min^−1^, 0.0202 min^−1^, and 0.0192 min^−1^ across five successive cycles. These results demonstrate the substantial reproducibility and operational stability of the photocatalyst. Conversely, the TOC removal kinetics constants *k_TOC_* obtained from similarly dynamic conditions through the cycles were 0.0231 min^−1^, 0.0205 min^−1^, 0.0192 min^−1^, 0.0178 min^−1^, and 0.0164 min^−1^. The slight decreasing trend in *k_TOC_* values across cycles indicates YGHP’s impressive recyclability and stability.

The recyclability and stability of Y_2_TmSbO_7_ and GdYBiNbO_7_ photocatalysts were assessed by examining AC degradation and TOC mineralization over five consecutive cycles under VLTE, as depicted in Figures S1 and S2. These figures clearly illustrate the structural stability of Y_2_TmSbO_7_ and GdYBiNbO_7_, indicating their potential for efficient recyclability in photodecomposition processes. Following 148 min of VLTE, the AC REY values for Y_2_TmSbO_7_ were measured as being 89.06%, 87.84%, 86.17%, 84.65%, and 83.05% over the successive cycles. Similarly, when utilizing GdYBiNbO_7_ as a photocatalyst, the AC REY values recorded were 82.19%, 80.97%, 79.33%, 77.81%, and 76.95% across the cycles. Additionally, the REY values of TOC achieved with Y_2_TmSbO_7_ as a photocatalyst remained consistently high, ranging from 85.16% to 79.29% across the respective cycles. Likewise, the REY values of TOC with GdYBiNbO_7_ as a photocatalyst ranged from 77.83% to 71.72% over the cycles. The above results confirm the recyclability and structural stability of Y_2_TmSbO_7_ and GdYBiNbO_7_ over multiple degradation cycles.

Moreover, *k_C_* and *k_TOC_* were analyzed during the photocatalytic degradation of AC utilizing Y_2_TmSbO_7_ and GdYBiNbO_7_ photocatalysts over five successive cycles under VLTE conditions, and the results are presented in Figures S1 and S2. The obtained *k_C_* values for AC degradation when using Y_2_TmSbO_7_ as a photocatalyst consistently exhibited high values across the five cycles, with calculated values of 0.0132 min^−1^, 0.0121 min^−1^, 0.0115 min^−1^, 0.0109 min^−1^, and 0.0103 min^−1^. Similarly, the *k_C_* values for AC degradation with GdYBiNbO_7_ as a photocatalyst also showed high values, calculated as being 0.0102 min^−1^, 0.0095 min^−1^, 0.0091 min^−1^, 0.0087 min^−1^, and 0.0084 min^−1^ across the five cycles. The above results demonstrate the eximious recyclability and stability of both Y_2_TmSbO_7_ and GdYBiNbO_7_ photocatalysts. In contrast, the obtained *k_TOC_* values for TOC removal during AC photodegradation with Y_2_TmSbO_7_ under dynamic conditions exhibited a decreasing trend across the cycles, with values of 0.0114 min^−1^, 0.0107 min^−1^, 0.0101 min^−1^, 0.0096 min^−1^, and 0.0091 min^−1^. Similarly, the *k_TOC_* values for TOC removal during AC photodegradation with GdYBiNbO_7_ also showed a slight decreasing trend, with values of 0.0089 min^−1^, 0.0084 min^−1^, 0.080 min^−1^, 0.0077 min^−1^, and 0.0073 min^−1^. The above results further highlight the impressive recyclability and stability of the Y_2_TmSbO_7_ and GdYBiNbO_7_ photocatalysts in achieving efficient TOC mineralization.

In addition, Figure S3 illustrates the impact of varying YGHP dosages on the removal efficiencies of AC under the condition of VLTE for 148 min. The highest REY of AC, reaching 99.75%, was observed at an initial concentration of YGHP of 0.8 g/L. However, subsequent increases in the initial concentration of YGHP led to a decline in the REY of AC. This decrease could be attributed to the aggregation of a high concentration of YGHP, resulting in reduced active sites on the catalyst’s surface.

Figure S4 presents the impact of varying pH values on the REY of AC using YGHP as the catalyst under VLTE conditions and darkness. Significantly, uniform degradation efficiencies towards AC were noted across pH values of 3, 7, and 11 following 148 min of VLTE. The results depicted in Figure S4 indicate AC degradation rates of 99.33%, 99.75%, and 98.79% for pH values of 3, 7, and 11, respectively, following VLTE. Furthermore, the results shown in Figure S4 revealed an equilibrium between detachment and attachment after 48 min of dark reaction, with no significant change in AC saturation observed under dark conditions for pH values of 3, 7, or 11. Therefore, the data indicate that different pH values had no discernible influence on the REY of AC using YGHP, either under VLTE or in darkness.

Moreover, the photodegradation experiment also investigated the REY of YGHP on parathion-methyl, isocarbophos, chlorpyrifos, and diuron. Figure S5 illustrates that following 148 min of visible light irradiation, YGHP exhibited removal rates of 99.75%, 97.81%, 96.51%, 81.88%, and 82.81% for AC, parathion-methyl, isocarbophos, chlorpyrifos, and diuron, respectively. These results further underscore the significant potential of YGHP in the purification of pesticide-contaminated wastewater.

**Figure 14:**
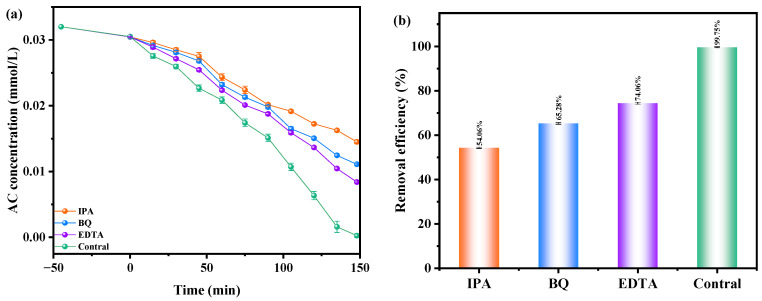
Impact of different radical scavengers on (**a**) AC saturation and (**b**) removal efficiency of AC.

Figure 14a,b show how the presence of benzoquinone (BQ), isopropanol (IPA), and ethylene diamine tetraacetic acid (EDTA) as radical inhibitors affected the degradation efficiency of AC when using YGHP as the catalyst under VLTE conditions. IPA, BQ, and EDTA were employed to eliminate •OH, •O_2_^−^, and h^+^ radicals, respectively, introduced at the onset of the photodecomposition process to identify the reactive species engaged in degradation. Following comparative analysis against the comparison group, it was observed that IPA caused an average decrease in AC degradation efficiency of 45.69%, while BQ and EDTA led to reductions of 34.47% and 25.69%, respectively. The findings suggest that •OH, •O_2_^−^, and h^+^ actively participate as radicals in the degradation process, with •OH showing the most effective oxidation removal ability. Therefore, in the presence of YGHP, •OH radicals efficiently eradicated AC in agrochemical wastewater. The order of effectiveness in removing AC through oxidation was determined to be •OH > •O_2_^−^ > h^+^.

**Figure 15:**
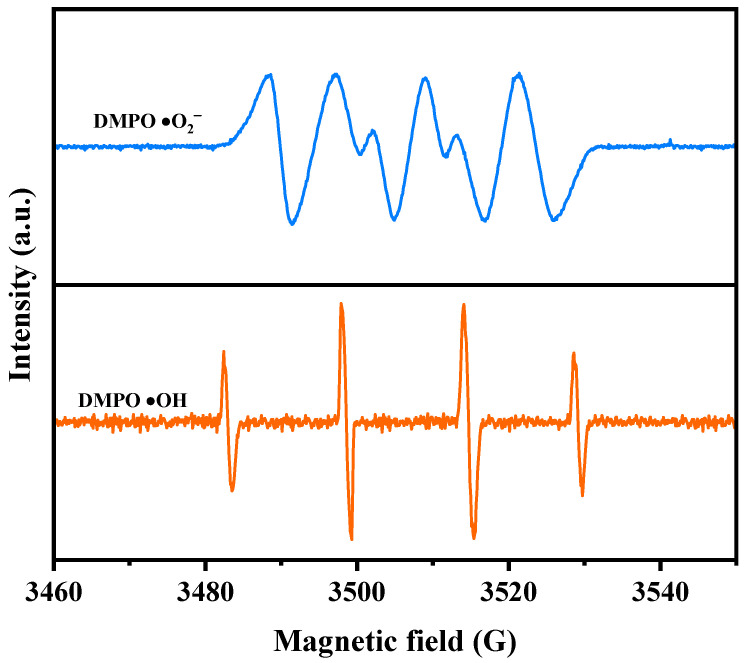
EPR spectrum for DMPO·O_2_^−^ and DMPO·OH over YGHP.

Furthermore, the generation of •O_2_^−^ and •OH during the photodecomposition process was subjected to electron paramagnetic resonance (EPR) analysis. Figure 15 illustrates the EPR spectrum of the signals for DMPO•O_2_^−^ and DMPO•OH in the presence of YGHP. After being exposed to visible light for 10 min, the EPR spectrum showed a distinct DMPO•O_2_^−^ signal containing four unambiguous peaks with an equal intensity ratio of 1:1:1:1, thus authenticating the existence of •O_2_^−^ radicals [77]. Subsequently, the EPR spectrum exhibited a signal containing four lines with an intensity ratio of 1:2:2:1, authenticating the existence of the DMPO•OH signal following VLTE [77]. Based on these findings, it is indicated that both •O_2_^−^ radicals and •OH radicals were generated concurrently during the photodecomposition process. Importantly, the EPR signals’ relative intensity indicates a greater production of •OH radicals in comparison to •O_2_^−^ radicals [77,78]. The outcomes of the radical scavenger experiments align with the results, providing tangible evidence for the participation of •O_2_^−^ and •OH in the degradation mechanism.

**Figure 16:**
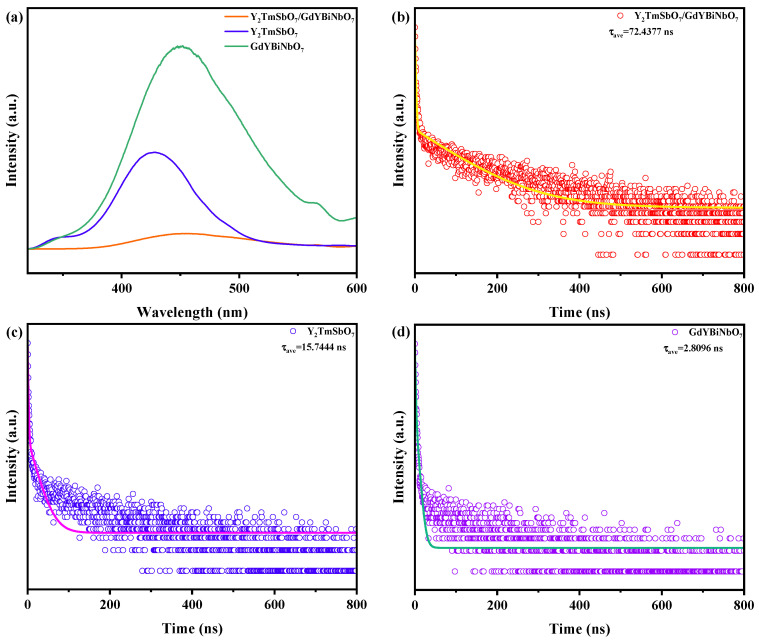
(**a**) PL spectrum of YGHP, Y_2_TmSbO_7_, and GdYBiNbO_7_, and TRPL spectra of (**b**) YGHP, (**c**) Y_2_TmSbO_7_, and (**d**) GdYBiNbO_7_.

Figure 16a–d present the photoluminescence (PL) and time-resolved photoluminescence (TRPL) spectra of Y_2_TmSbO_7_, GdYBiNbO_7_, and YGHP. Increased PL intensities are indicative of accelerated recombination rates of PECs, resulting in reduced photocatalytic efficiency. Of all the samples, YGHP showed the least photometric intensity, indicating effective charge separation and restricted recombination of PECs [79,80]. Y_2_TmSbO_7_ had a higher photometric intensity, while GdYBiNbO_7_ showed the highest photometric intensity. These results indicate the improved photocatalytic performance of the heterostructure sample for degrading AC. Furthermore, further confirmation was achieved to substantiate the stronger photocatalytic efficacy of YGHP in comparison to Y_2_TmSbO_7_ and GdYBiNbO_7_. A double-exponential decay equation (3) was utilized to obtained the fitting results of the TRPL spectra shown in Figure 16b–d [81]:

(3) $$I(t)=I_{0}+A_{1}exp(-\frac{t}{\tau_{1}})+A_{2}exp(-\frac{t}{\tau_{2}})$$

As per the provided equation, *A*_1_ and *A*_2_ represent the respective weighting coefficients of the first- and second-order decay times for each decay channel [82]. Equation (4) was utilized to reckon the average PECs lifetime ($\tau_{ave}$) [83]:

(4) $$\tau_{ave}=(A_{1}\tau_{1}^{2}+A_{2}\tau_{2}^{2})/(A_{1}\tau_{1}+A_{2}\tau_{2})$$

The lifetimes that were calculated, along with the respective parameters, are listed in Table 3. On account of the formation of the fact that the heterojunction could facilitate the separation of PECs, YGHP showed markedly prolonged lifetimes ($\tau_{1}$ = 1.4762 ns, = 103.8215 ns, = 72.4377 ns) compared to Y_2_TmSbO_7_ ($\tau_{1}$ = 19.5543 ns, = 1.1976 ns, = 15.7444 ns) and GdYBiNbO_7_ ($\tau_{1}$ = 1.2731 ns, = 5.5767 ns, = 2.8096 ns). The above findings confirm the unparalleled ascendency of YGHP in photocatalytic effectiveness, exceeding that of Y_2_TmSbO_7_ and GdYBiNbO_7_.

**Figure 17:**
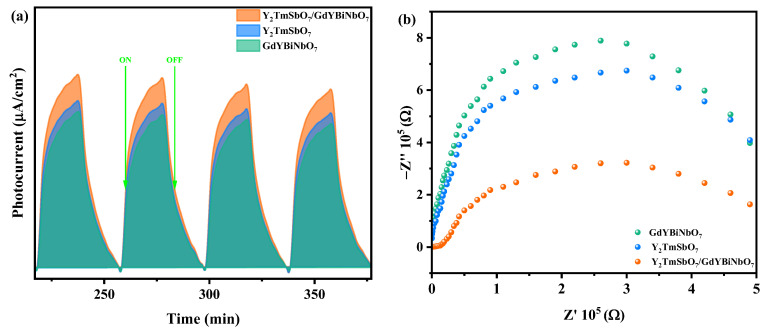
(**a**) Transient photocurrent and (**b**) EIS plots of YGHP, Y_2_TmSbO_7_, and GdYBiNbO_7_.

The photocatalytic performance of the synthesized YGHP, Y_2_TmSbO_7_, and GdYBiNbO_7_ samples was assessed in terms of the efficiency of PEC separation and the enhancement of interfacial charge transfer behavior. The investigation was conducted through a combination of photocurrent (PC) and electrochemical impedance spectroscopy (EIS) techniques. Figure 17a reveals that YGHP exhibited a higher photocurrent density than the individual Y_2_TmSbO_7_ and GdYBiNbO_7_ samples. This finding indicates that the integration of Y_2_TmSbO_7_ and GdYBiNbO_7_ may have promoted the separation of PECs [84,85]. The consistent photocurrent intensity without significant fluctuations suggests robust stability and reproducibility of the sample’s photocatalytic activity. To corroborate these findings, EIS Nyquist plots were employed, as shown in Figure 17b. A smaller radius in a Nyquist plot typically signifies lower charge transfer resistance [86,87]. YGHP indeed demonstrated a smaller radius compared to Y_2_TmSbO_7_ and GdYBiNbO_7_, suggesting a more efficient separation of PECs. This observation aligns with the results of the PL and time-resolved TRPL spectra, as well as transient photocurrent responses. Altogether, these results verify that the heterojunction between Y_2_TmSbO_7_ and GdYBiNbO_7_ distinctly accelerates the separation of photogenerated electrons and holes, thereby prolonging the lifetime of PECs.

**Figure 18:**
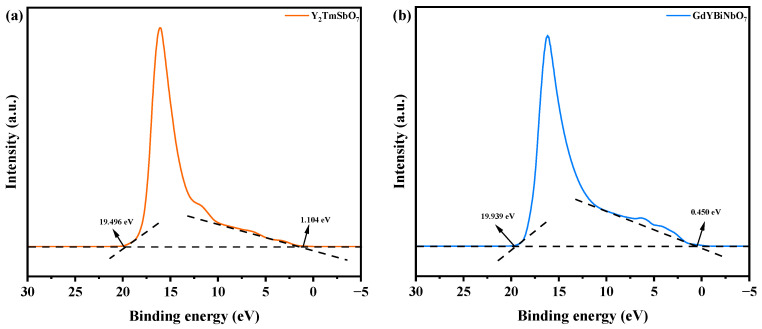
UPS spectra of (**a**) Y_2_TmSbO_7_ and (**b**) GdYBiNbO_7_ (the intersections of the black dash lines indicated by the black arrows indicated the onset (Ei) and cutoff (Ecutoff) binding energy).

Figure 18 displays the ultraviolet photoelectron spectroscopy (UPS) spectra of Y_2_TmSbO_7_ and GdYBiNbO_7_, providing valuable information for the determination of their ionization potentials. The measured onset (Ei) and cutoff (Ecutoff) binding energies shown in Figure 18 demonstrate values of 1.104 eV and 19.496 eV for Y_2_TmSbO_7_, and 0.450 eV and 19.939 eV for GdYBiNbO_7_ [88]. By considering the excitation energy (approximately 21.2 eV), the ionization potentials of Y_2_TmSbO_7_ and GdYBiNbO_7_ were accurately determined as being 2.808 eV and 1.711 eV, respectively [89,90]. Consequently, it was estimated that the conduction band (CB) band potentials for Y_2_TmSbO_7_ and GdYBiNbO_7_ were 0.153 eV and −0.692 eV, respectively.

**Figure 19:**
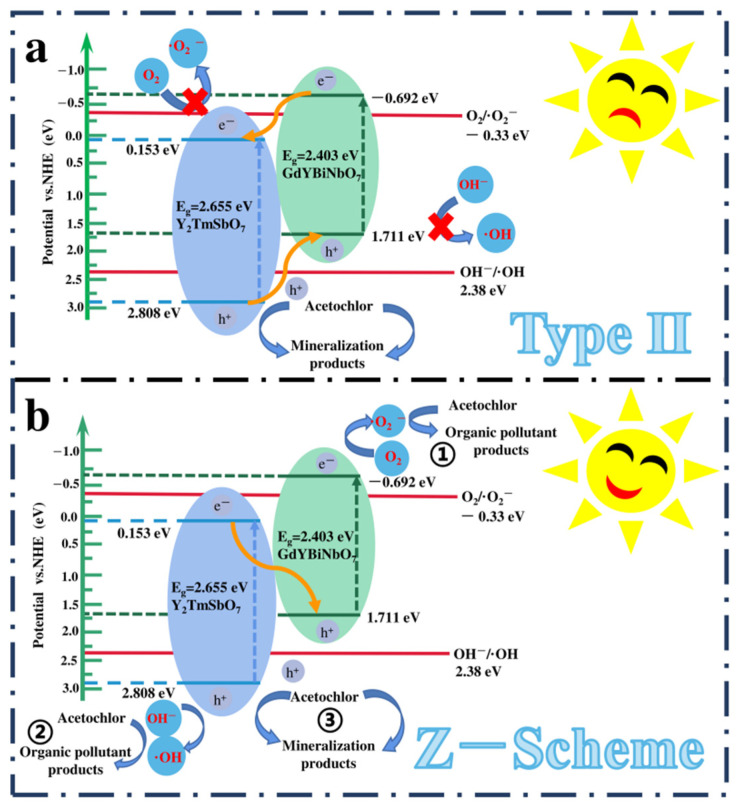
Plausible photodegradation mechanism of AC with YGHP as photocatalyst under VLTE: (**a**) conventional type II and (**b**) direct Z-scheme.

Following the examination of the CB and valance band (VB) positions of Y_2_TmSbO_7_ and GdYBiNbO_7_, this research delved deeply into the photocatalytic mechanism of YGHP, presenting two plausible modes: the conventional type-II mode and the direct Z-scheme heterojunction [91], as illustrated in Figure 19. In the traditional type-II transfer mode (Figure 19a), photoexcited electrons from the CB of GdYBiNbO_7_ were theorized to transfer to the CB of Y_2_TmSbO_7_. Simultaneously, photoexcited holes from the VB of Y_2_TmSbO_7_ would migrate to the VB of GdYBiNbO_7_. Under this assumption, the electrons in the CB of Y_2_TmSbO_7_ would not effectively react with O_2_ to generate •O_2_^−^ due to the more positive E_CB_ of Y_2_TmSbO_7_ (0.153 eV) compared to the O_2_/•O_2_^−^ potential (−0.33 eV vs. NHE) [92]. Similarly, the generation of •OH in the VB of GdYBiNbO_7_ would be inhibited by the negative E_VB_ value of GdYBiNbO_7_ (1.711 eV), which was lower than the OH^−^/•OH potential (2.38 eV vs. NHE) [93,94]. However, these findings contradicted the experimental detection results of •O_2_^−^ and •OH radicals in trapping experiments and EPR analyses.

Hence, the direct Z-scheme heterojunction type (as depicted in Figure 19b) appeared to offer a more suitable explanation for the enhanced photocatalytic mechanism observed in this study. In the Z-scheme configuration, photogenerated electrons were anticipated to migrate from the CB of Y_2_TmSbO_7_ (0.153 eV) to the VB of GdYBiNbO_7_ (1.711 eV), thereby facilitating the direct recombination of the PECs derived from the inferior oxidation–reduction potentials within the heterostructure. This mechanism maintained high reduction and oxidation potentials essential for photocatalytic reactions. Therefore, this configuration enabled electrons in the CB of GdYBiNbO_7_ (−0.692 eV) to interact with O_2_, forming •O_2_^−^ species, crucial for decomposing AC (① in Figure 19b). Similarly, the holes occupying the VB of Y_2_TmSbO_7_ (2.808 eV) were anticipated to react with OH^−^, resulting in the generation of •OH species, which play a crucial role in the degradation of AC (② in Figure 19b). Moreover, the holes residing in either the VB of Y_2_TmSbO_7_ or GdYBiNbO_7_ could potentially function as catalytic reactive radicals for the oxidation and subsequent degradation of AC owing to their inherent strong oxidation potential (③ in Figure 19b). Consequently, the generated active species of •O_2_^−^, •OH, and h^+^ would contribute to the elimination of AC, which was consistent with the findings of trapping experiments and EPR tests. Based on the aforementioned analysis, it is evident that the direct Z-scheme heterojunction provides a more suitable explanation for the enhanced photocatalytic mechanism observed in the Y_2_TmSbO_7_/GdYBiNbO_7_ heterojunction. The above elucidation not only clarifies the photodegradation pathways but also highlights the robust potential of the Y_2_TmSbO_7_/GdYBiNbO_7_ heterostructure in environmental remediation applications, marking a significant advancement in the field of AC photodegradation.

**Figure 20:**
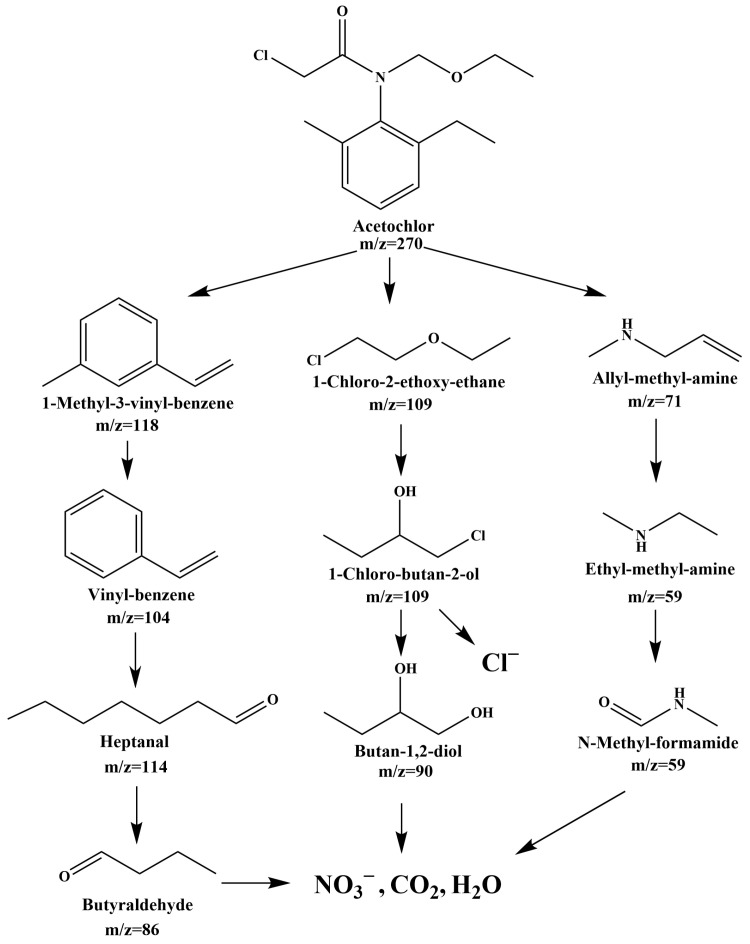
Feasible photodegradation pathway scheme for AC under VLTE with YGHP as catalyst.

To elucidate the degradation pathway of AC, previous investigations and LC-MS were employed to analyze the intermediates generated during the degradation procedure [41,95]. In light of the gathered data, a feasible photocatalytic degradation pathway for AC was put forward, as illustrated in Figure 20. Initially, the N-C bond linked to the benzene ring in AC underwent cleavage due to the attack of •OH, yielding degradation products such as 1-methyl-3-vinyl-benzene (*m*/*z =* 118), allyl-methyl-amine (*m*/*z =* 71), and 1-chloro-2-ethoxy-ethane (*m*/*z =* 109) [41]. Subsequently, oxidative reactions led to further transformations of these intermediates. For instance, the methyl group in 1-methyl-3-vinyl-benzene was oxidized by •OH to form vinyl-benzene (*m*/*z =* 104) [41]. Further oxidative reactions fractured the benzene ring and the C=C bond in vinyl-benzene, yielding heptanal (*m*/*z =* 114) [41]. Additionally, alkane groups at the chain end were oxidized by •OH, resulting in the production of butyraldehyde (*m*/*z =* 86) [41]. Ultimately, •OH facilitates the conversion of intermediates into CO_2_ and H_2_O. Allyl-methyl-amine reacted with •OH, undergoing oxidation to form ethyl-methyl-amine (*m*/*z =* 59) [41]. The continuous oxidation of ethyl-methyl-amine led to the formation of N-methyl-formamide (*m*/*z =* 59) [41], which was further oxidized to NO_3_^−^, CO_2_, and H_2_O. Moreover, 1-chloro-2-ethoxy-ethane (*m*/*z =* 109) underwent oxidation and rearrangement, yielding 1-chloro-butan-2-ol (*m*/*z =* 109) [41]. Hydrolysis of 1-chloro-butan-2-ol resulted in the production of Cl^−^ and butane-1,2-diol (*m*/*z =* 90) [41], which was subsequently oxidized to CO_2_ and H_2_O.

## 3. Experimental Section

### 3.1. Materials and Reagents

All chemicals were utilized in the original state, without undergoing additional purification: Gd(NO_3_)_3_·6H_2_O (Merck Co., Ltd., Shanghai, China, 99.999%); Bi(NO_3_)_3_·5H_2_O (Merck Co., Ltd., Shanghai, China, 99.999%); Tm(NO_3_)_3_·5H_2_O (Merck Co., Ltd., Shanghai, China, 99.9%); benzoquinone (BQ, C_6_H_4_O_2_, Merck Co., Ltd., Shanghai, China, ≥99.5%); Y(NO_3_)_3_·6H_2_O (Macklin Biochemical Co., Ltd., Shanghai, China, 99.99%); NbCl_5_ (Macklin Biochemical Co., Ltd., Shanghai, China, 99.999%); SbCl_5_ (Macklin Biochemical Co., Ltd., Shanghai, China, 99.999%); ethylenediaminetetraacetic acid (EDTA, C_10_H_16_N_2_O_8_, Macklin Biochemical Co., Ltd., Shanghai, China, 99.99%); pure ethanol (C_2_H_5_OH, Macklin Biochemical Co., Ltd., Shanghai, China, 99.5%); octanol (C_8_H_18_O, Macklin Biochemical Co., Ltd., Shanghai, China, 99.5%); ethylene glycol (C_2_H_6_O_2_, Macklin Biochemical Co., Ltd., Shanghai, China, 99%); acetochlor (AC, C_14_H_20_ClNO_2_, Macklin Biochemical Co., Ltd., Shanghai, China, 99.5%); and isopropyl alcohol (IPA, C_3_H_8_O, Aladdin Group Chemical Reagent Co., Ltd., Shanghai, China, ≥99.999%).

### 3.2. Preparation Method of Y_2_TmSbO_7_

The Y_2_TmSbO_7_ photocatalytic materials were prepared using the hydrothermal synthesis technique in this investigation [96,97]. In this study, in comparison to solid phase sintering, the hydrothermal synthesis method offered several advantages, including the ability to operate under lower temperature and pressure conditions. This resulted in the production of materials with uniformity and high purity, as well as the ability to control morphology. Furthermore, the hydrothermal synthesis method provided a milder chemical reaction environment. The precursor materials Y(NO_3_)_3_·6H_2_O, Tm(NO_3_)_3_·5H_2_O, SbCl_5_ and nitric acid were employed to synthesize Y_2_TmSbO_7_ with a stoichiometric ratio of Y/Tm/Sb of 2:1:1. An equal amount of Y(NO_3_)_3_·6H_2_O (2.4 mol/L), Tm(NO_3_)_3_·5H_2_O (1.2 mol/L), and SbCl_5_ (1.2 mol/L) were mixed completely, then the resulting precursor substances were placed into a reaction container (high-pressure autoclave). A mixture of glycerol and water was employed as the reaction medium, with the addition of ethylene glycol to aid in dispersion. This solution filled 60% of the autoclave’s volume. Subsequently, the reaction vessel was positioned in a high-temperature furnace and raised to a temperature of 240 °C. The autoclave was pressurized to 150 MPa and the reaction proceeded for 1400 min before being cooled to ambient temperature. The pressure inside the autoclave underwent significant changes during the heating and insulation processes. Specifically, as the temperature increased during the heating process, the liquid in the solution evaporated into steam, leading to an increase in pressure within the autoclave. Upon reaching a heating temperature of 240 °C, the pressure inside the autoclave reached approximately 150 MPa and remained constant at this level throughout the thermal insulation reaction. Following a 1400 min heat retention period, heating was discontinued, and the autoclave was gradually cooled to room temperature, causing the pressure to return to ambient levels. The resulting mixture underwent centrifugal filtration and was then sequentially washed with acetone, deionized water, and pure ethanol. Afterward, the mixture was evaporated at room temperature under vacuum. Ultimately, to ensure a more uniform phase formation of the material, the resulting powder mixture was compacted into pellets and sintered in a furnace under the following conditions: starting from room temperature, the temperature was ramped up to 920 °C with a ramping time of 130 min, followed by a dwell time of 720 min, and finally cooled down to room temperature. The resulting pellet was crushed, resulting in the successful synthesis of pure Y_2_TmSbO_7_. Additionally, the visual diagram of the preparation and synthesis of Y_2_TmSbO_7_ is shown in Figure S6.

### 3.3. Preparation Method of GdYBiNbO_7_

The hydrothermal synthesis technique was also employed for the preparation of the GdYBiNbO_7_ photocatalytic material [96,97]. Equal volumes of Gd(NO_3_)_3_·6H_2_O (1.2 mol/L), Y(NO_3_)_3_·6H_2_O (1.2 mol/L), Bi(NO_3_)_3_·5H_2_O (1.2 mol/L), and NbCl_5_ (1.2 mol/L) were used as precursor materials. The autoclave was heated to 220 °C and maintained at this temperature for 1600 min. The heating procedure in the high-temperature furnace involved gradually increasing the temperature from room temperature to 960 °C over 180 min, followed by a dwell time of 850 min, and concluded with a gradual cooling within the furnace. To obtain the desired pure GdYBiNbO_7_, the resulting pellet underwent crushing. To clarify the preparation technology, the visual diagram of the preparation and synthesis of GdYBiNbO_7_ is shown in Figure S7.

### 3.4. Synthesis of N-Doped TiO_2_

N-TiO_2_ was synthesized by combining a hydrolysis co-precipitation method with a nitrogen atmosphere calcination process. Tetrabutyl titanate was dissolved in deionized water with a molar ratio of 1:30 between tetrabutyl titanate and water. The hydrolysis reaction was carried out under magnetic stirring at 1200 r/min. Following a 450 min stirring period, the mixture was then filtered using suction to separate the particles, and subsequently dried in an oven at 80 °C for 24 h. Subsequently, the powdered particles were loaded into a tube furnace and calcined at 550 °C under a nitrogen atmosphere for 90 min to obtain N-TiO_2_.

### 3.5. Synthesis of Y_2_TmSbO_7_/GdYBiNbO_7_ Heterojunction Photocatalyst

YGHP was prepared via ultras-assisted hydrothermal synthesis. Equal quantities of Y_2_TmSbO_7_ and GdYBiNbO_7_ prepared via hydrothermal synthesis were mixed in octanol and sonicated for 150 min in an ultrasonic bath. The mixture was subsequently vigorously stirred and maintained at 180 °C for 240 min to encourage the bonding of Y_2_TmSbO_7_ onto the surface of GdYBiNbO_7_ nanoparticles, forming Y_2_TmSbO_7_/GdYBiNbO_7_ heterostructure catalytic materials. Once cooled to room temperature, the product was retrieved using centrifugation and underwent multiple washes with ethanol. The clarified powder was evaporated in a vacuum oven at 80 °C for 270 min and then kept in a desiccator for future usage. Ultimately, the Y_2_TmSbO_7_/GdYBiNbO_7_ heterostructure catalytic materials were successfully prepared. The schematic representation of the preparation and synthesis of YGHP is shown in Figure S8.

Furthermore, following the preparation method described above, the estimated costs of N-T, GdYBiNbO_7_, Y_2_TmSbO_7_, and YGHP were approximately USD 353.5 per 100 g, USD 33.6 per 100 g, USD 31.4 per 100 g, and USD 32.4 per 100 g, respectively. It is evident that the cost of N-T was 10.5 times higher than that of GdYBiNbO_7_, 11.3 times higher than that of Y_2_TmSbO_7_, and 10.9 times higher than that of YGHP.

### 3.6. Characterization

Crystallographic information was obtained using X-ray diffraction (XRD) with an XRD-6000 diffractometer from Shimadzu Corporation in Kyoto, Japan. Microstructural and morphological features were examined through transmission electron microscopy (TEM) using the Talos F200X G2 TEM from Thermo Fisher Scientific in Waltham, MA, USA. The elemental composition was determined using energy-dispersive spectroscopy (EDS). UV-Vis diffuse reflectance spectrophotometry (UV-Vis DRS) was used to study the optical properties of the samples, conducted with a UV-3600 spectrophotometer from Shimadzu Corporation in Kyoto, Japan. Functional groups and chemical bonding were examined using a WQF-530A spectrometer from Beifen-Ruili Analytical Instrument (Group) Co., Ltd., located in Beijing, China, for Fourier transform infrared spectroscopy (FTIR) analysis. Raman spectrometry was conducted to investigate chemical bond interactions using an INVIA0919-06 instrument from RENSHAW plx, located in Wotton-under-Edge, Gloucestershire, GL12 8JR, London, United Kingdom. X-ray photoelectron spectroscopy (XPS) was performed using a PHI 5000 VersaProbe instrument from UlVAC-PHI in Maoqi City, Japan, to examine surface chemical composition and states. Ultraviolet photoelectron spectroscopy (UPS) measurements were carried out to determine the ionization potential of the valence band using an Escalab 250 xi instrument from Thermo Fisher Scientific, located in Waltham, MA, USA. Additionally, electron paramagnetic resonance (EPR) spectroscopy was utilized to detect information about the presence of free radicals in the samples, employing an A300 instrument from Bruker Corporation, located in Karlsruhe, Germany. The properties of PECs were determined using an FLS980 spectrophotometer from Edinburgh Instruments Ltd., located in Edinburgh, United Kingdom.

### 3.7. Photoelectrochemical Experiments

EIS tests were performed utilizing a CHI660D electrochemical workstation from Chenhua Instruments Co., Ltd., located in Shanghai, China. The fabricated materials served as the working electrode, while a platinum plate and an Ag/AgCl electrode were used as the counter and reference electrodes, respectively. A 1.5 mol/L Na_2_SO_4_ aqueous solution was employed as the electrolyte. For the preparation of the working electrode, the prepared photocatalytic material sample weighing 3 mg was mixed with 300 μL of ethanol and 7.5 μL of Nafion reagent, followed by ultrasonic dispersion for 2.5 h. Afterward, the obtained suspension was carefully transferred using a micropipette and evenly spread onto the conductive surface of a glassy carbon electrode. Finally, the sample was allowed to air-dry naturally.

### 3.8. Experimental Setup and Procedure

The degradation studies were carried out in a photocatalytic reactor (CEL-LB70, manufactured by China Education Au-Light Technology Co., Ltd., based in Beijing, China) under visible light conditions. Figures S9 and S10 showcase the outside and inside diagrams of the photocatalytic reactor, respectively. The light was produced using a 500 W xenon lamp and a 420 nm cutoff filter. For each experiment, 12 quartz tubes were utilized, with each tube containing 35 mL of the reaction solution. In the case of pesticide wastewater, the overall reaction volume was 420 mL. The nanophase materials (Y2TmSbO7, GdYBiNbO7 or YGHP) were applied at a dosage of 0.8 g/L for the photodegradation experiment. The initial concentration of the specific compound in the solution was measured to be 0.032 mmol/L.

Throughout the degradation reaction, 5 mL samples of the solution were collected at regular intervals for subsequent analysis. Following 148 min of exposure to light, a 15 mL sample of the solution was obtained to assess the remaining AC concentration. The photocatalytic materials were removed through filtration using a 0.22 µm PES (polyether sulfone) filter membrane. Subsequently, the remaining AC saturation was assessed via an Agilent 200 high-performance liquid chromatography system (Agilent Technologies, Palo Alto, CA, USA). During the post-photodegradation phase of the AC dispersion system, a 10 µL volume was injected at a flow rate of 1 mL/min. Following this, the degradation system, which included the photocatalytic nanomaterials and AC, was stirred in darkness for 45 min before exposure to light. This allowed the system to reach adsorption/desorption equilibrium. The degradation system was stirred at a speed of 800 r/min under visible light conditions.

To evaluate the mineralization of AC in the dispersed liquid, a TOC analyzer (TOC-5000 A, Shimadzu Corporation, Kyoto, Japan) was employed. For the purpose of calibrating the TOC saturation during the AC photodecomposition process, either potassium acid phthalate (KHC8H4O4) or anhydrous sodium carbonate was utilized as the reference reagent. Standards for calibration were created, with established carbon concentrations ranging from 0 to 100 mg/L, utilizing potassium acid phthalate. The evaluation of TOC saturation was conducted for twelve samples, each consisting of a reaction solution volume of 35 mL.

To recover photocatalysts between cycles, the reaction solution was transferred to a centrifuge tube for subsequent centrifugation following the completion of the photocatalytic reaction. The tube was subjected to high-speed centrifugation at 1200 r/min, effectively precipitating the solid photocatalyst to the bottom of the tube. Upon photocatalyst precipitation, the supernatant was carefully decanted to avoid disturbing the precipitated photocatalyst. The resulting precipitate was then subjected to further centrifugation using pure water or ethanol to remove any remaining reactants or products on the photocatalyst. Subsequently, the washed photocatalyst underwent another round of centrifugation to ensure thorough cleaning, after which the washing solvent was removed. The collected photocatalyst was then transferred to a drying dish or an appropriate container for the removal of residual moisture or solvent. Ultimately, the dried photocatalyst was ready for characterization or for utilization in subsequent rounds of photocatalytic experiments.

To conduct calibration experiments on intermediate reactants, Thermo Quest LCQ Duo (Thermo Fisher Scientific Corporation, Waltham, MA, USA) liquid chromatography-mass spectrometry (LC-MS) was used. In the AC photocatalytic process, a Beta Basic-C18 HPLC column (Thermo Fisher Scientific Corporation, Waltham, MA, USA) was employed. Following the photocatalytic reaction, a 20 µL solution generated from the reaction was automatically injected into the LC-MS system. The mobile phase utilized in the LC-MS system was a 60% methanol and 40% ultrapure water mixture. The analysis was configured with a mass-to-charge ratio (*m*/*z*) ranging from 50 to 400.

In order to measure the intensity of the light irradiation, a filter with a wavelength of 420 nm was employed to specifically select the desired visible light range of radiation. The calculation of the number of photons that pass through the filter per unit time, whether total photons or reactive photons, involves using the formula υ = c/λ. Within this formula, υ stands for the photon frequency, λ represents the wavelength of the incident light, and c indicates the velocity of light. Additionally, the energy of a photon (hν) could be precisely determined by utilizing the specific numerical values of the Avogadro constant (NA) and Planck constant (h). The flux of irradiating photons could be modified by altering the separation between the photoreactor and the light source. A radiometer (Model FZ-A, Photoelectric Instrument Factory Beijing Normal University, Beijing, China) was employed to quantify the incident photon flux, *I*_o_. The specific value recorded during light exposure was 4.76 × 10^−6^ Einstein L^−1^ s^−1^. The PEY was calculated utilizing Equation (5):

*ϕ* = *R*/*I_o_* (5)

T *ϕ* represents the PEY (%), *R* denotes the RV of AC (mol/L/s), and *I_o_* represents the irradiation photon flux (Einstein L^−1^ s^−1^).

## 4. Conclusions

This work presents a groundbreaking synthesis of the direct Z-scheme YGHP utilizing an ultrasound-assisted hydrothermal synthesis technique, alongside the fabrication of novel photocatalytic nanomaterials, Y_2_TmSbO_7_ and GdYBiNbO_7_, through the use of the hydrothermal fabrication technique. The characterization of these materials involved a comprehensive suite of techniques, including XRD, UV-Vis DRS, FTIR spectroscopy, Raman spectroscopy, XPS, TEM-EDS, PC test, EIS, UPS, PL spectroscopy, and EPR spectroscopy. Furthermore, YGHP exhibited eximious photocatalytic activity, achieving a 99.75% degradation efficiency of AC and a 97.90% mineralization efficiency of TOC within 148 min of VLTE. Comparative analysis demonstrated that YGHP displayed significantly higher photocatalytic performance for AC compared to the Y_2_TmSbO_7_, GdYBiNbO_7_, or N-T photocatalysts, with removal efficiencies being 1.12 times, 1.21 times, or 3.07 times higher, respectively. Meanwhile, successive degradation cycle experiments affirmed the structural robustness and sustained efficacy of YGHP. The highly enhanced photocatalytic performance could be credited to the formation of a Z-scheme heterojunction structure between Y_2_TmSbO_7_ and GdYBiNbO_7_, enhancing PEC transfer rates and maintaining higher redox potentials. Moreover, trapping experiments and EPR tests certified the involvement of •O_2_^−^, •OH, and h^+^ in the photocatalytic degradation of AC. Additionally, a credible degradation pathway and mechanism for AC were proposed. In conclusion, this study significantly contributes to the field of photocatalysis by offering promising strategies for the treatment of AC-contaminated pesticide wastewater, advancing the understanding of Z-scheme heterojunction photocatalytic systems, and providing insights for the development of effective Z-scheme heterojunction photocatalysts.

## Figures and Tables

**Table 1:** Configurable properties of Y_2_TmSbO_7_ fabricated using hydrothermal fabrication technique.

Atom	x	y	z	Occupation Factor
Y	0	0	0	1
Tm	0.5	0.5	0.5	0.5
Sb	0.5	0.5	0.5	0.5
O(1)	−0.178	0.125	0.125	1
O(2)	0.125	0.125	0.125	1

**Table 2:** Configurable properties of GdYBiNbO_7_ fabricated using hydrothermal fabrication technique.

Atom	x	y	z	Occupation Factor
Gd	0	0	0	0.5
Y	0	0	0	0.5
Bi	0.5	0.5	0.5	0.5
Nb	0.5	0.5	0.5	0.5
O(1)	−0.170	0.125	0.125	1
O(2)	0.125	0.125	0.125	1

**Table 3:** Fitted results of TRPL curves of Y_2_TmSbO_7_, GdYBiNbO_7_, and YGHP.

	Y_2_TmSbO_7_	GdYBiNbO_7_	Y_2_TmSbO_7_/GdYBiNbO_7_
$A_{1}$	0.0393	0.8945	0.9518
$\tau_{1}$ (ns)	19.5543	1.2731	1.4762
$A_{2}$	0.9589	0.1134	0.0306
$\tau_{2}$ (ns)	1.1976	5.5767	103.8215
$\tau_{ave}$ (ns)	15.7444	2.8096	72.4377

## Data Availability

Data are contained within the article.

## Supplementary Materials

The following supporting information can be downloaded at: https://www.mdpi.com/article/10.3390/ijms25136871/s1.
