# Supplementary material for: Constructing Direct Z-Scheme Y2TmSbO7/GdYBiNbO7 Heterojunction Photocatalyst with Enhanced Photocatalytic Degradation of Acetochlor under Visible Light Irradiation
Source: Int J Mol Sci. 2024 Jun 22;25(13):6871. doi: 10.3390/ijms25136871 (PMC11241117; doi:10.3390/ijms25136871)
Supplement: Supplementary file 1 [file ijms-25-06871-s001.zip › ijms-3036329-supplementary.pdf]

Constructing Direct Z-Scheme $Y_2TmSbO_7/GdYBiNbO_7$ Heterojunction Photocatalyst with Enhanced Photocatalytic Degradation of Acetochlor under Visible Light Irradiation

Liang Hao [1] and Jingfei Luan [1,2,*]

[1] School of Physics, Changchun Normal University, Changchun 130032, China; hliang0725@163.com
[2] State Key Laboratory of Pollution Control and Resource Reuse, School of the Environment, Nanjing University, Nanjing 210093, China
* Correspondence: jfluan@nju.edu.cn; Tel.: +86-19951939498

Table S1. Comparison of photocatalytic efficacy of YGHP with other reported photocatalysts in photodegradation process of AC.

**Photocatalyst**	**Radiation**	**Irradiation time (min)**	**Pesticide**	**Removal rate (%)**	**Ref**
α-Fe_2O_3 hollow nanoparticles	UV	180	Acetochlor	91	[41]
BiOBr	Visible light	480	Acetochlor	13.76	[7]
g-C_3N_4	Visible light	480	Acetochlor	82.75	[7]
g-C_3N_4/BiOBr-ethanol	Visible light	480	Acetochlor	91	[7]
g-C_3N_4/BiOBr-ethylene glycol	Visible light	480	Acetochlor	77	[7]
Oxidized carbon nitrides	Visible light	180	Acetochlor	≤90	[42]
YGHP	Visible light	148	Acetochlor	99.75	This study

Table S2. Comparison of the photocatalysts prepared in this study and the photocatalysts reported in our previous studies with the photocatalytic effect of AC.

Photocatalyst	Radiation	Irradiation time (min)	Pesticide	Removal rate (%)
YGHP	Visible light	148	Acetochlor	99.75
Y_2TmSbO_7	Visible light	148	Acetochlor	89.06
$GdYBiNbO_7$	Visible light	148	Acetochlor	82.19
Er_2FeSbO_7	Visible light	148	Acetochlor	69.36
$Er_2FeSbO_7/BiTiSbO_6$ heterojunction photocatalyst	Visible light	148	Acetochlor	75.65
Bi_2SmSbO_7	Visible light	148	Acetochlor	64.57
$Bi_2SmSbO_7/ZnBiYO_4$ heterojunction photocatalyst	Visible light	148	Acetochlor	73.44
In_2YSbO_7	Visible light	148	Acetochlor	75.32
$In_2YSbO_7/BiSnSbO_6$ heterojunction photocatalyst	Visible light	148	Acetochlor	83.01
Gd_2BiTaO_7	Visible light	148	Acetochlor	66.34
Ag_3PO_4/Gd_2BiTaO_7 heterojunction photocatalyst	Visible light	148	Acetochlor	74.51

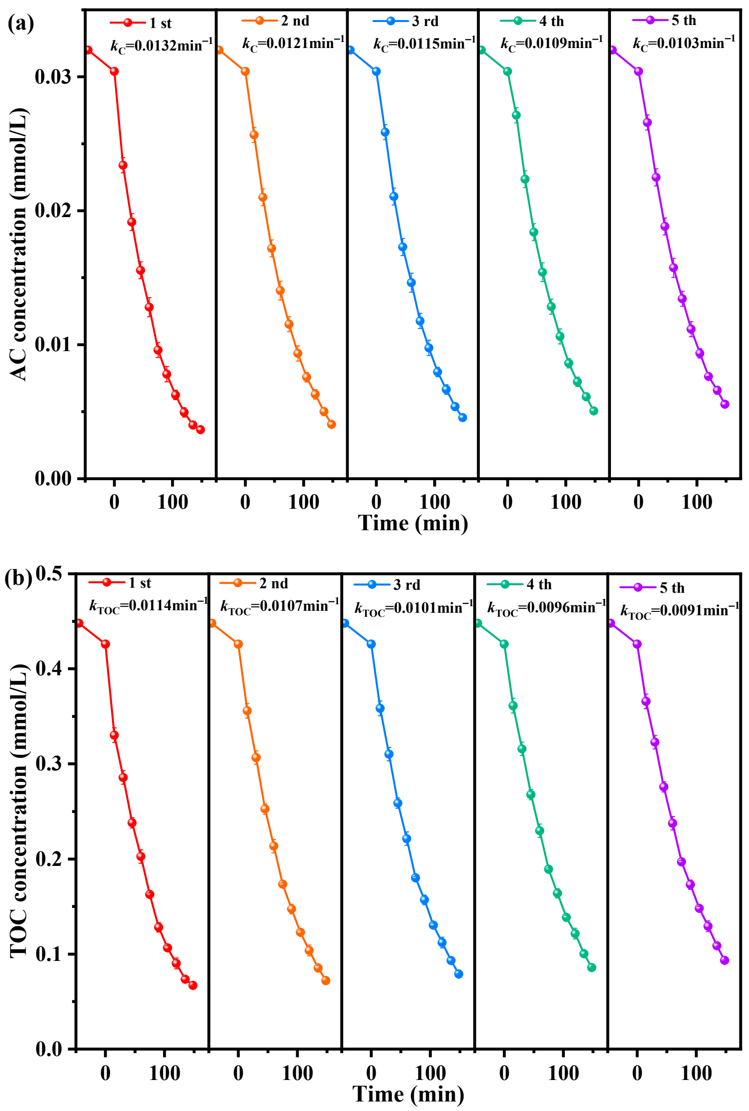

Figure S1. Saturation fluctuation imageries of **(a)** AC and **(b)** TOC during photodegradation of AC in pesticides wastewater with Y_2TmSbO_7 as photocatalyst under VLTE for successive degradation trials.

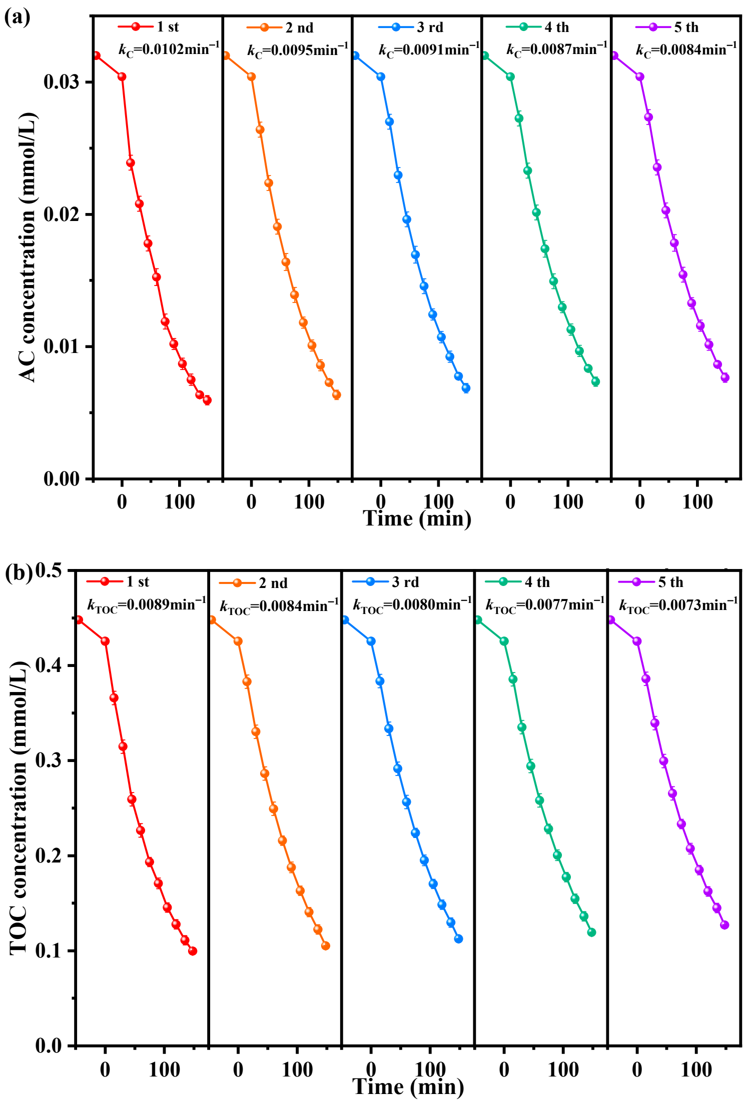

Figure S2. Saturation fluctuation imageries of **(a)** AC and **(b)** TOC during photodegradation of AC in pesticides wastewater with $GdYBiNbO_7$ as photocatalyst under VLTE for successive degradation trials.

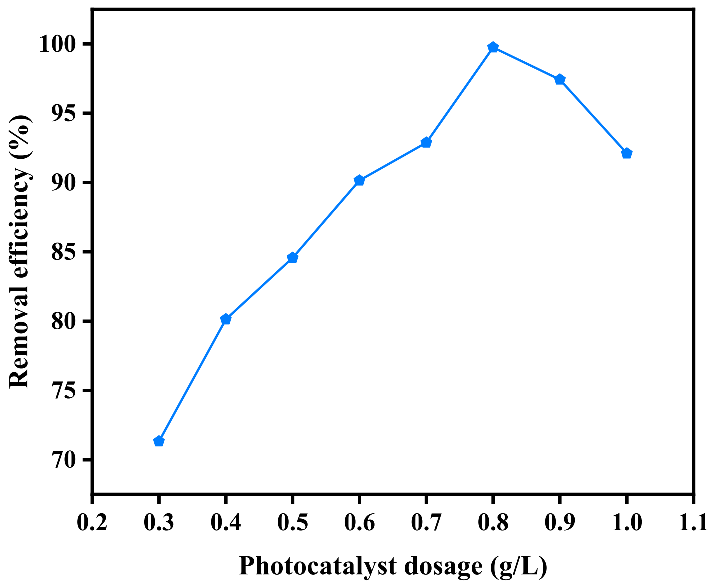

Figure S3. Impact of YGHP dosage on removal efficiency of AC.

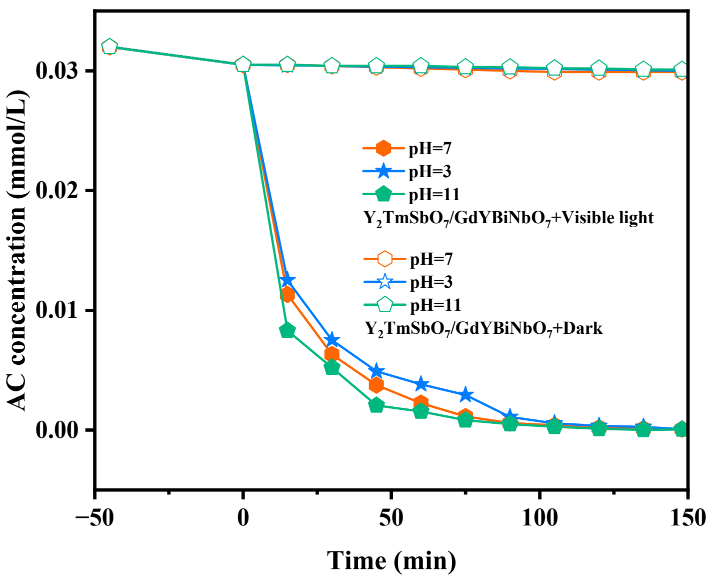

Figure S4. The effect of different pH values on AC degradation with YGHP as catalyst under VLTE.

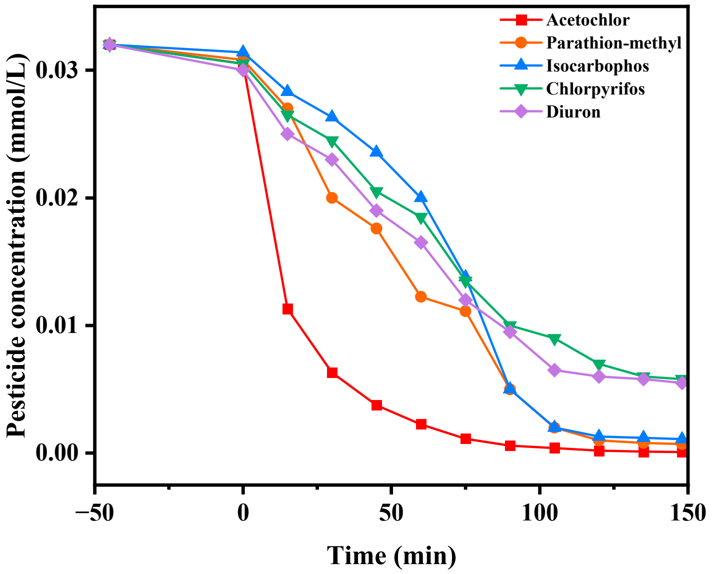

Figure S5. Saturation fluctuation charts of AC, parathion-methyl, isocarbophos, chlorpyrifos, and diuron with YGHP as the catalytic sample under VLTE.

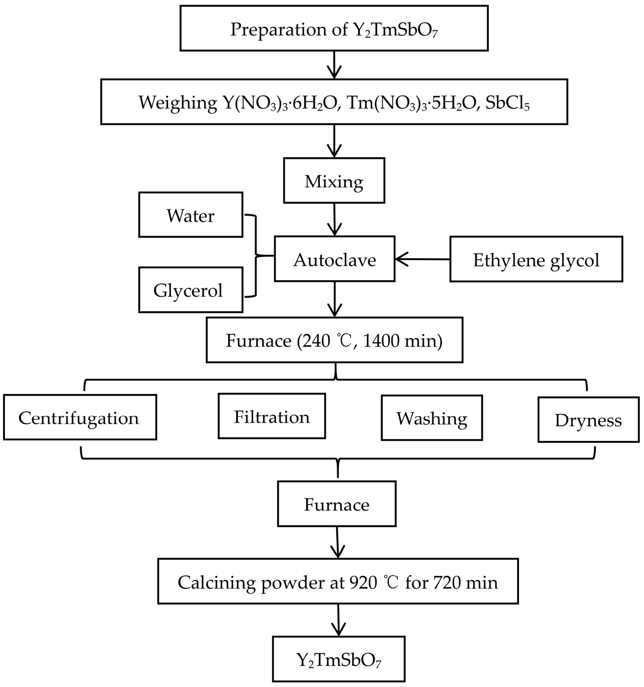

Figure S6. Schematic representation of the synthesis process of the Y_2TmSbO_7.

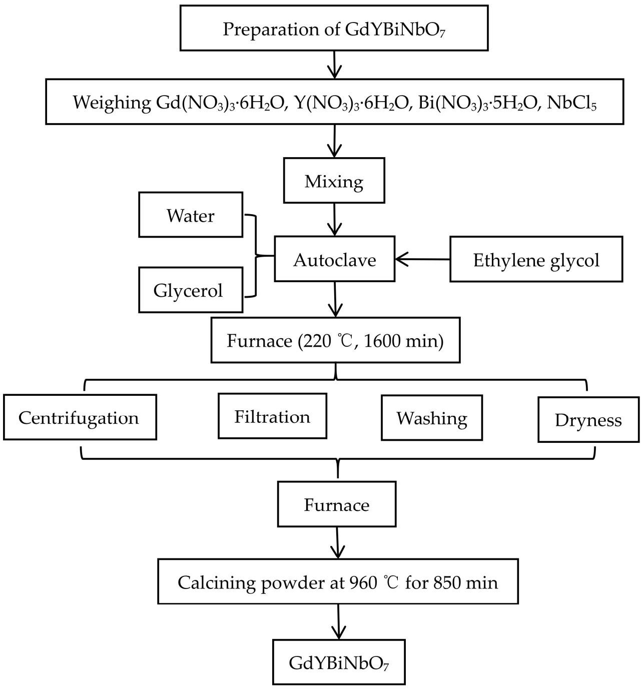

Figure S7. Schematic representation of the synthesis process of the $GdYBiNbO_7$.

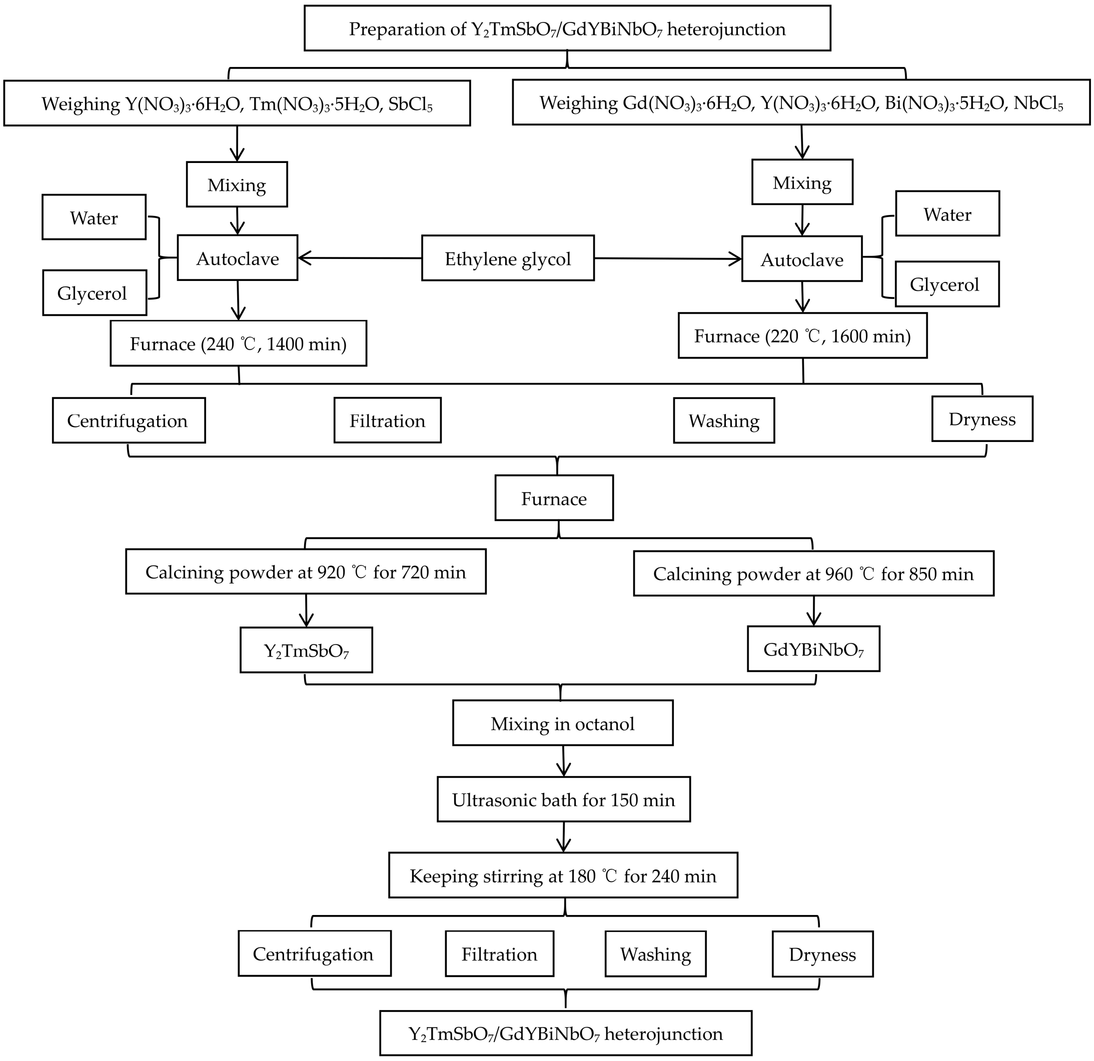

Figure S8. Schematic representation of the synthesis process of the YGHP.

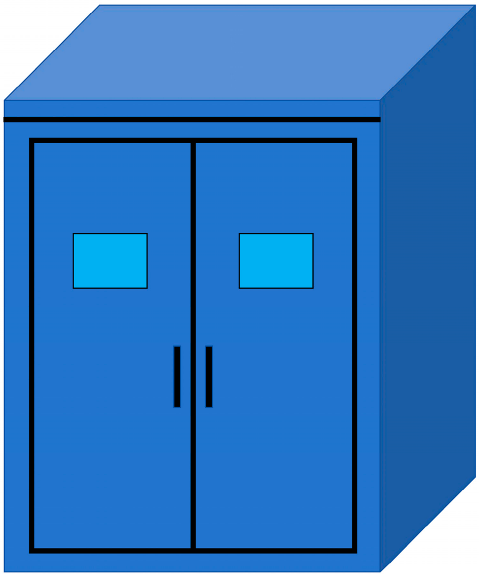

Figure S9. Brief diagram of the photocatalytic reactor (outside).

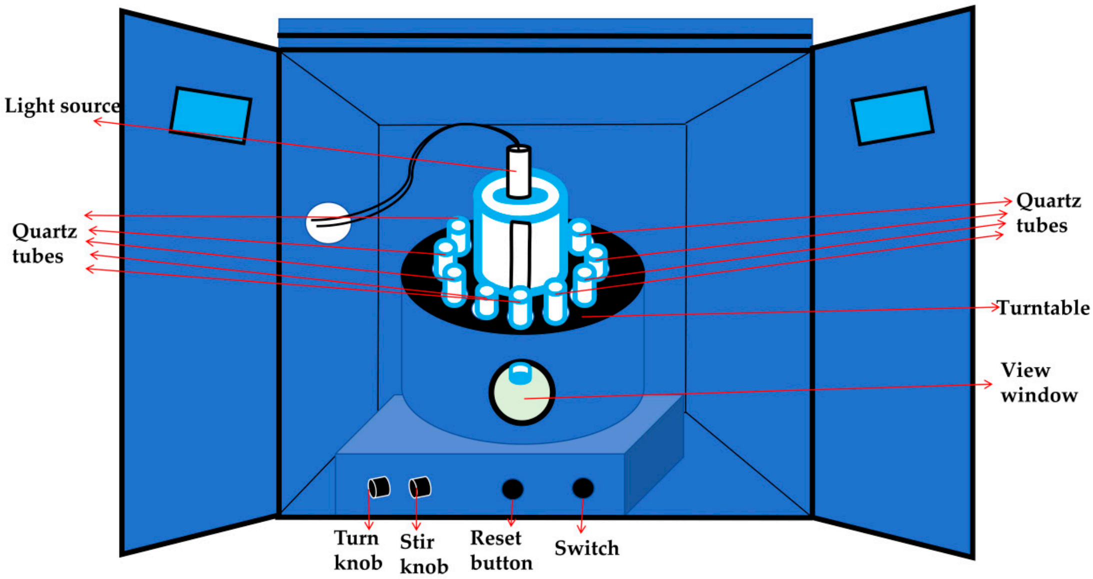

Figure S10. Brief diagram of the photocatalytic reactor (inside).
